# Photoluminescence and Scintillation Mechanism of Cs_4_PbBr_6_

**DOI:** 10.1021/acs.jpcc.4c06347

**Published:** 2024-11-12

**Authors:** J. Jasper van Blaaderen, Andries van Hattem, Jence T. Mulder, Daniel Biner, Karl W. Krämer, Pieter Dorenbos

**Affiliations:** † Faculty of Applied Sciences, Department of Radiation Science and Technology, Delft University of Technology,, Mekelweg 15, 2629 JB Delft, The Netherlands; ‡ Faculty of Applied Sciences, Optoelectronic Materials Section, Delft University of Technology,, Van der Maasweg 9, 2629 HZ Delft, The Netherlands; § Department of Chemistry, Biochemistry, and Pharmaceutical Sciences, Univeristy of Bern, Freiestrasse 3, 3012 Bern, Switzerland

## Abstract

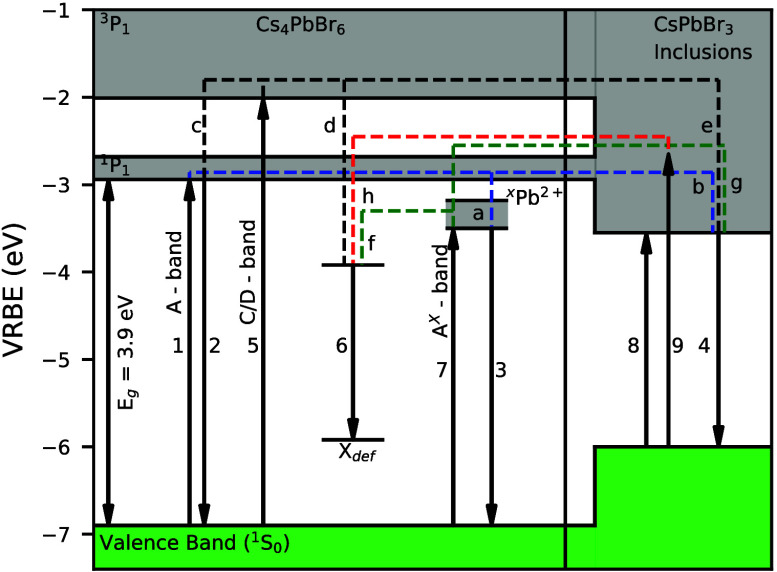

Small bandgap scintillators have gained significant attention in recent years. Especially Cs_4_PbBr_6_ is an interesting material, mitigating the small Stokes shift-related problem of perovskites like CsPbBr_3_. In this work, optical and scintillation properties of Cs_4_PbBr_6_ single crystals are investigated as a function of temperature, with a detailed focus at 10 K. The Cs_4_PbBr_6_ single crystals were grown using the vertical Bridgman method. Due to incongruent melting, CsPbBr_3_ inclusions are formed, generating a 540 nm emission band. Prepairing Cs_4_PbBr_6_ via solid-state synthesis yields CsPbBr_3_-inclusion-free material, showing no green 540 nm emission band. In Cs_4_PbBr_6_ samples with and without CsPbBr_3_ inclusions, a new emission band at 610 nm ascribed to an unknown defect was found. Based on the presented experiments, an emission mechanism is proposed for Cs_4_PbBr_6_. This shows that both defects and CsPbBr_3_ inclusions play a role in the emission behavior of Cs_4_PbBr_6_ but only the CsPbBr_3_ inclusions are responsible for the 540 nm emission.

## I Introduction

In the last 10 years, lead halide perovskites^1−3^ and lead halide perovskite-related compounds^4−6^ have gained interest in the field of X-ray and γ photon scintillation detection. A clear distinction should be made, as discussed by Akkerman and Manna, between true perovskites and perovskite-related compounds.^7^ The perovskite crystal structure, with stoichiometry ABX_3_, consists of a three-dimensional network of corner-sharing BX_6_ octahedra. The small bandgap of lead halide perovskites, approximately 3 eV, increases their theoretical maximum light yield compared to larger bandgap traditional scintillators.^1,8,9^ Using eq 1 and a bandgap of 3 eV, it can be estimated that perovskites could surpass 100 000 photons/MeV scintillation photon yield.
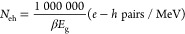
1Here, *N*_eh_ represents the number of created electron–hole pairs, β is taken to be ≈2.5, and *E*_g_ represents the bandgap of the scintillator in eV. Perovskite-based scintillators differ from traditional scintillators, often doped or codoped, by being intrinsic scintillators, i.e., not relying on dopant ions.^10−14^

One of the shortcomings of lead halide perovskite-based scintillators, however, is their small Stokes shift, which results in losses due to self-absorption.^3,15,16^ This problem has been addressed in detail by Wolszczak et al.^15^ and Williams et al.^16^ For other applications, like light-emitting diodes, this is a significantly smaller problem; these applications often use either thin films or nanocrystals.^17−19^ Potential solutions to deal with the small Stokes shift of perovskites are lower-dimensional lead halide compounds or the introduction of a dopant to downshift the emission wavelength. Dimension, in this case, refers to the connectivity of the PbX_6_ octahedral network. In the genuine perovskite structure, ABX_3_, the PbX_6_ network is conected in three dimensions. The dimensionality decreases to two-dimensional in compounds like (PEA)_2_PbBr_4_ or zero-dimensional in compounds like Cs_4_PbBr_6_. Lower-dimensional compounds often show self-trapped exciton emission which, compared to the near bandgap free exciton emission of perovskites, has a significantly larger Stokes shift.^20−23^

Examples of lower-dimensional lead-based compounds that have been studied for their scintillation properties are the two-dimensional hybrid organic–inorganic perovskites phenethylammonium lead bromide ((PEA)_2_PbBr_4_), butylammonium lead bromide ((BA)_2_PbBr_4_), and 2,2-(ethylenedioxy)bis(ethylammonium) lead chloride ((EDBE)PbCl_4_).^1,4,24,25^ In these compounds, the cation located on the A site is replaced with a relatively large ammonium ion molecule, creating an alternating layered structure of PbX_6_ octahedra and organic layers.

In this work, the low-temperature optical and scintillation properties of the zero-dimensional lead halide compound Cs_4_PbBr_6_ are studied. Cs_4_PbBr_6_ crystallizes in the K_4_CdCl_6_ structure with space group *R*3̅*c*,^26,27^ has a density of 4.19 g/cm^3^, and a 3.9 eV bandgap.^28−30^ Compared to the corner-sharing network of BX_6_ octahedra in the perovskite crystal structure, the A_4_BX_6_ structure consists of isolated BX_6_ octahedra.^31,32^ This results in the formation of localized states, composed of the 6s^2^ and 6s6p states of Pb^2+^, on the BX_6_ octahedra. This closely resembles low doping concentrations of Pb^2+^ in face-centered cubic (fcc) alkali halide crystals, also resulting in the formation of isolated [PbX_6_]^4–^ octahedra.^29,33^

**Figure 1:**
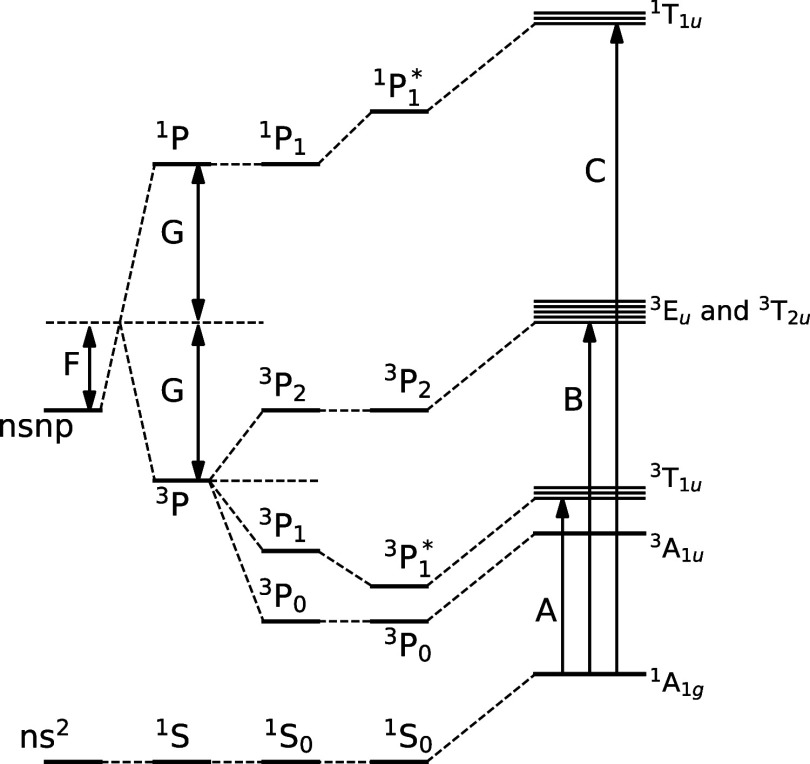
Schematic energy level diagram of ions with an ns^2^ electronic ground state. Here, F represents the shift of the energy levels due to electrostatic interactions and G represents the shift due to exchange interactions. The final formation of the A-, B-, and C-bands and the corresponding symmetries are shown for an octahedrally surrounded ns^2^ ion. The * indicates the mixing of the ^3^*P*_1_ and ^1^*P*_1_ levels due to spin–orbit interaction.

A general schematic of the energy levels of ions with an ns^2^ electronic configuration, e.g., Tl^+^, Pb^2+^, and Bi^3+^, is shown in Figure 1. The energy levels of a free ns^2^ ion shift and split due to a combination of electrostatic, exchange, and spin–orbit interactions.^34,35^ In a crystal field, the levels will split further forming three distinct absorption bands, labeled the A (^1^*S*_0_ → ^3^*P*_1_), B (^1^*S*_0_ → ^3^*P*_2_), and C (^1^*S*_0_ → ^1^*P*_1_) band, which correspond with transitions between the ns^2^ ground state and nsnp excited states.^34,35^ The ^1^*S*_0_ → ^1^*P*_1_ transition, or the C-band, is allowed. The ^1^*S*_0_ → ^3^*P*_0_, ^1^*S*_0_ → ^3^*P*_1_ (A-band), and ^1^*S*_0_ → ^3^*P*_2_ (B-band) transitions are spin forbidden. The ^1^*S*_0_ → ^3^*P*_1_ transition, or A-band, becomes partially allowed due to the spin–orbit coupling mixing the ^3^*P*_1_ and ^1^*P*_1_ states.^34^ The ^1^*S*_0_ → ^3^*P*_2_ transition, or B-band, can be induced due to vibrational coupling with the crystal lattice but it remains weak and often escapes observation.^35,36^ Additionally, a fourth band, labeled the D-band, corresponding to a metal to metal charge transfer state between the ground state of Pb^2+^ and the conduction band is often observed.^37,38^ Emission typically takes place due to transitions from the ^3^*P*_1_ (excited) state to the ^1^*S*_0_ (ground) state and is commonly referred to as A-band emission.^34,35^

Cs_4_PbBr_6_ has been studied in different morphologies: nanocrystals,^28,39−42^ thin films,^29,30,43^ single crystals,^27,44,45^ and powders.^46,47^ Next to the potential interest of Cs_4_PbBr_6_ as a scintillator, it has also gained interest to be used in light-emitting diodes (LEDs),^48,49^ luminescent solar concentrates,^50,51^ and UV detectors.^52^ Additionally, Cs_4_PbBr_6_ has been explored as host matrix for the production of CsPbBr_3_ nanocrystals, an overview of which is presented by Akkerman et al.^52^ Cs_4_PbBr_6_ especially gained a lot of interest due to its intense green 540 nm emission in addition to its UV 380 nm emission. The origin of the 540 nm emission is sometimes still under debate. It was suggested to either originate from bromine vacancies^27,41,53−55^ or CsPbBr_3_ nanocrystal inclusions.^29,30,45,56−60^ The latter is seen as the more favorable explanation. This debate has recently been summarized in the review articles of Wang et al.,^61^ Biswas,^62^ and Akkerman et al.^52^ In general, two different types of Cs_4_PbBr_6_ can be classified in the literature: Cs_4_PbBr_6_ with CsPbBr_3_ inclusions, showing UV 380 nm and green 540 nm emission, and phase pure Cs_4_PbBr_6_, showing only UV 380 nm emission. The goal of this work is to propose a general emission mechanism for Cs_4_PbBr_6_, by studying the low-temperature optical and scintillation properties of Cs_4_PbBr_6_. The experiments are performed on a Cs_4_PbBr_6_ single crystal, grown using the vertical Bridgman method. One of the problems of producing single crystals from the melt, however, is the incongruent melting of Cs_4_PbBr_6_.^63^ This results in the formation of CsPbBr_3_ inclusions.^29,30^ Such inclusions have also been observed in CsBr crystals doped with Pb^2+^.^64−66^ Zhang et al. demonstrated that Cs_4_PbBr_6_ single crystals can be grown from solution without CsPbBr_3_ inclusions in the presence of an excess of Cs^+^ and Br^–^ ions, which stabilize the formation of Cs_4_PbBr_6_.^57^ Akkermann et al. have demonstrated a similar approach for the production of Cs_4_PbBr_6_ nanocrystals.^28^ We have chosen to use a solid-state synthesis, next to the Bridgman-grown single crystal, to produce a Cs_4_PbBr_6_ powder without CsPbBr_3_ inclusions. The solid-state reaction between CsBr and PbBr_2_ takes place below the incongruent melting point of Cs_4_PbBr_6_.^63^ A slight excess of CsBr was chosen to prevent the formation of CsPbBr_3_ inclusions. This approach has also been used for the synthesis of Cs_4_PbI_6_.^67^

**Figure 2:**
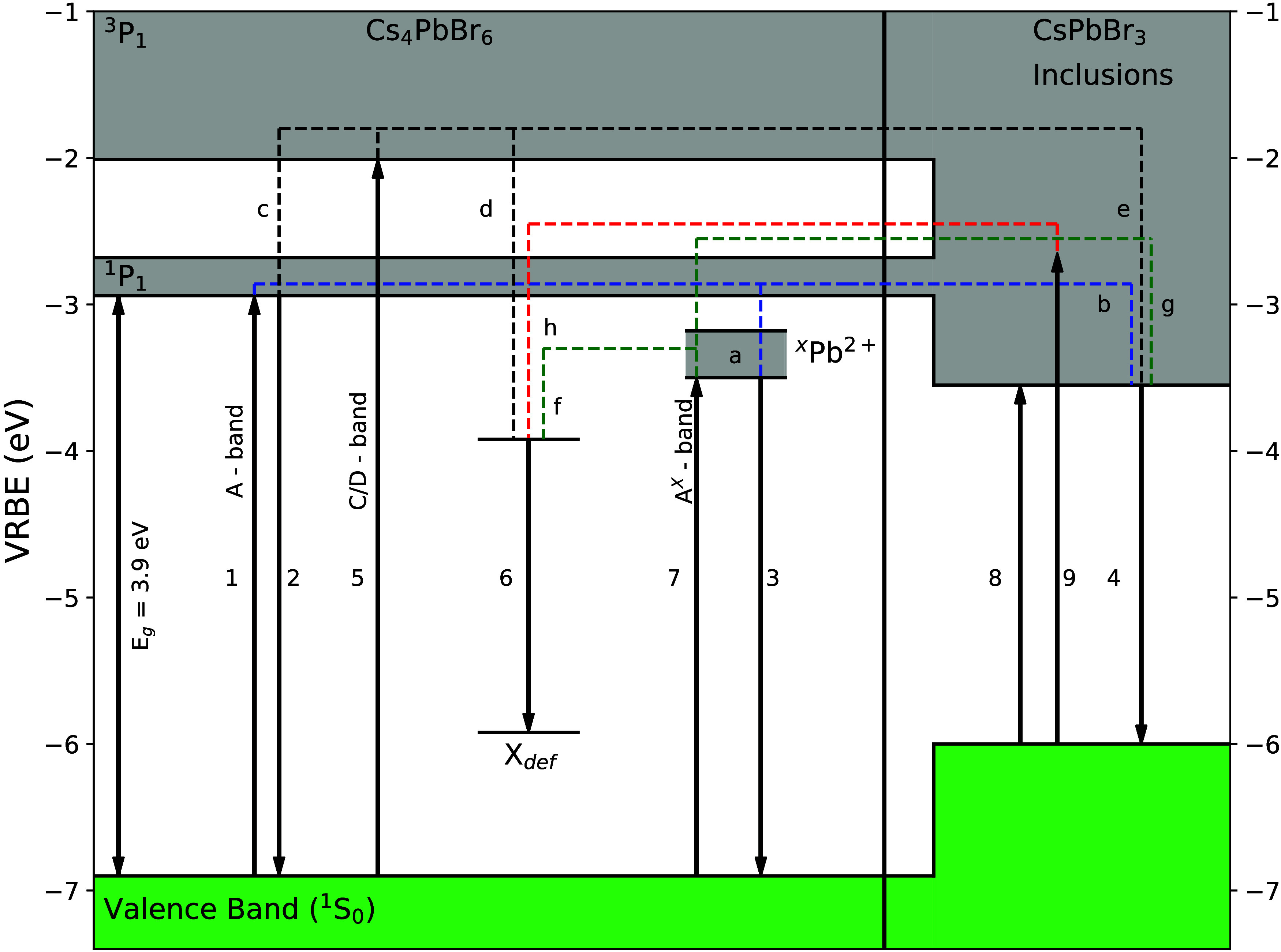
Vacuum referred binding energy (VRBE) diagram and the processes taking place in Cs_4_PbBr_6_ on the left and the VRBE plus additional processes taking place in Cs_4_PbBr_6_ with CsPbBr_3_ inclusions on the right. Excitation and emission processes are represented by arrows and labeled using numbers. Energy transfer processes are indicated by dashed lines and labeled using letters. *X*_def_ represents the defect-related emission, and ^*x*^Pb^2+^ represents Pb^2+^ ions with a perturbed coordination shell.

In order to aid the presentation of the results and discussion presented in this work, Figure 2 shows the vacuum referred binding energy (VRBE) diagram applicable to Cs_4_PbBr_6_ on the left side and to CsPbBr_3_ on the right side. The valence band of Cs_4_PbBr_6_ consists of bromine 4p orbitals and lead 6s orbitals while the conduction band consists of bromine 5p orbitals and lead 6s6p orbitals.^41,57,68^ Vertical arrows represent excitation and emission transitions identified in the Section III. The horizontal dashed lines illustrate the energy and/or charge carrier transfer routes proposed in the Section IV.

## II Experimental Section

Crystals of Cs_4_PbBr_6_ were grown by the vertical Bridgman technique in sealed silica ampules. Since Cs_4_PbBr_6_ melts incongruently at 500 °C,^63^ a nonstoichiometric mixture of 75% CsBr and 25% PbBr_2_ was used. CsBr (Fluka, >99.5%) and PbBr_2_ (α, 5N) were dried at 200 °C in vacuum. In a drybox, the starting materials were filled into a silica ampule and sealed off under vacuum. The mixture was molten at 550 °C and slowly cooled by moving the furnace within 10 days. The product contained a white tip of CsBr and Cs_4_PbBr_6_, a yellow-orange middle part of Cs_4_PbBr_6_, and an orange top part of Cs_4_PbBr_6_ and CsPbBr_3_ eutecticum. Cs_4_PbBr_6_ crystals from the middle part were used for spectroscopic characterizations. Powder X-ray diffraction (Figure S1) and spectroscopic data revealed that the sample (middle part) contained about 90% Cs_4_PbBr_6_ and 10% CsPbBr_3_. The phase separation in the CsBr-PbBr_2_ melt did not work well, as can also be seen from the formation of a white tip containing CsBr.

CsBr (99.999%, Sigma-Aldrich) and PbBr_2_ (>98%, Alfa Aesar) were mixed in a 4.024–1.000 ratio and ground for 15 min. The mixture was loaded in a closed crucible and heated for 48 h at 600 K, after which it was let to cool down to room temperature. The as-obtained powder was loaded in an X-ray diffraction sample holder closed with Kapton foil to prevent powder spreading and reaction with moisture. The sample was measured on a PANalytical X’Pert PRO using Cu Kα-radiation (45 kV, 40 mA) in the range 10° < 2θ < 120° with an increment of 0.008° for a total measurement time of 9 h. The pattern was recorded using an X’Celerator detector. The obtained diffraction pattern was analyzed using Rietveld profile refinement^69,70^ in the FullProf suite.^71,72^ The analyzed powder diffraction pattern contained only the peaks of Cs_4_PbBr_6_ (>99%) and CsBr (<1%) as determined using Rietveld refinement.

X-ray excited emission spectra were recorded using a tungsten anode X-ray tube operated at 79 kV. This produces X-rays with an average energy of 40 keV. The low-energy part of the produced X-ray spectrum was removed by placing a 3 mm aluminum filter in front of the X-ray tube. This prevented radiation damage. The samples were mounted on the coldfinger of a closed cycle helium cryostat operated below 10^–4^ bar.

Pulsed X-ray excited emission spectra were recorded by using the time-correlated single-photon counting method. The start signal of the measurements was generated by a PicoQuant LDH-P-C440 M pulsed laser, which directly excites a Hamamatsu N5084 light-excited X-ray tube. This results in the production of X-ray pulses with an average energy of 18.2 keV. The stop signal of the measurements was generated by the detection of a single photon by an ID Quantique id100-50 single-photon counter. Both start and stop signals were processed by an Ortec 567 time-to-amplitude converter whose output signal was digitized by an Ortex AD 144 16K ADC. The samples were mounted on the coldfinger of a closed cycle helium cryostat operated below 10^–4^ bar.

Photoluminescence excitation and emission spectra were recorded by using light from a 450 W xenon lamp passing through a Horiba Gemini 180 monochromator to excite the sample. The emitted light was collected at a 90° angle with respect to the excitation source. The emitted light passed through a Princeton Instruments SpectraPro-SP2358 monochromator before being detected by a Hamamatsu R7600u-20. Any reflected excitation light was removed by using a long-pass filter in front of the Princeton monochromator. All spectra were corrected for the lamp intensity. The samples were mounted on the coldfinger of a closed cycle helium cryostat operated below 10^–4^ bar.

The presented absorbance spectra were recorded using a PerkinElmer Lambda 1050 instrument equipped with an integrating sphere.

## III Results

### III.I Cs_4_PbBr_6_ with CsPbBr_3_ Inclusions

**Figure 3:**
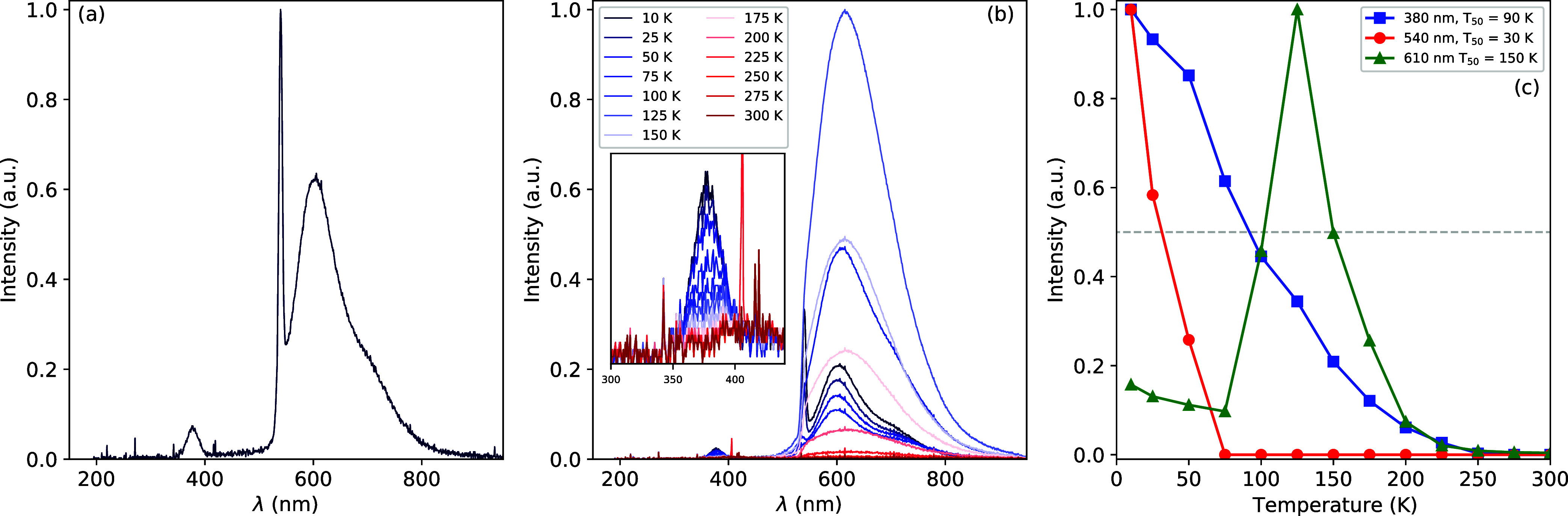
(a) X-ray excited emission spectrum of a Cs_4_PbBr_6_ single crystal with CsPbBr_3_ inclusions recorded at 10 K. (b) Temperature-dependent X-ray excited emission spectra of a Cs_4_PbBr_6_ single crystal measured from 10 to 300 K. The inset shows a zoom in of the 380 nm emission peak. (c) Temperature-dependent integrated emission intensities of the 380, 540, and 630 nm emission bands.

Figure 3a shows the 10 K X-ray excited emission spectrum of the Cs_4_PbBr_6_ single crystal. The spectrum contains three emission bands at 380, 540, and 610 nm. These emission bands have also been observed by Kubota et al.^73^ and Ding et al.^74^ Wu et al. only studied the emission at wavelengths longer than 400 nm under X-ray excitation, hence only observing the 540 and 610 nm emissions.^75,76^ The relative integral spectral intensities of the three emission bands are 1, 4.8, and 40.6, respectively. This means that the majority of the produced scintillation photons are present in the 610 nm emission band. The 380 nm UV band can be assigned to the intrinsic emission of Cs_4_PbBr_6_ (arrow 2 in Figure 2).^28,30,57,73^ The powder X-ray diffraction pattern of the Cs_4_PbBr_6_ sample is shown in Figure S1 shows that the single crystal contains CsPbBr_3_ related impurities to which the 540 nm emission can be ascribed (arrow 4 in Figure 2). It has been suggested by Kubota et al. that the 610 nm emission could be related to bromine vacancies (arrow 6 in Figure 2).^73−75^

The X-ray excited emission spectra, measured as a function of temperature, are shown in Figure 3b. The integrated intensities of the 380, 540, and 610 nm bands are shown in Figure 3c. The 540 nm emission band is fully quenched above 100 K. The intensity of the 610 nm emission shows a mild decrease until 75 K. Above 75 K, its intensity increases, reaching a maximum at 125 K, and then starts to quench. Based on the integrated peak intensities, the temperature (*T*_50_) at which the intensity drops below 50% of its maximum intensity for the 380, 540, and 610 nm emissions is 90, 30, and 150 K, respectively.

**Figure 4:**
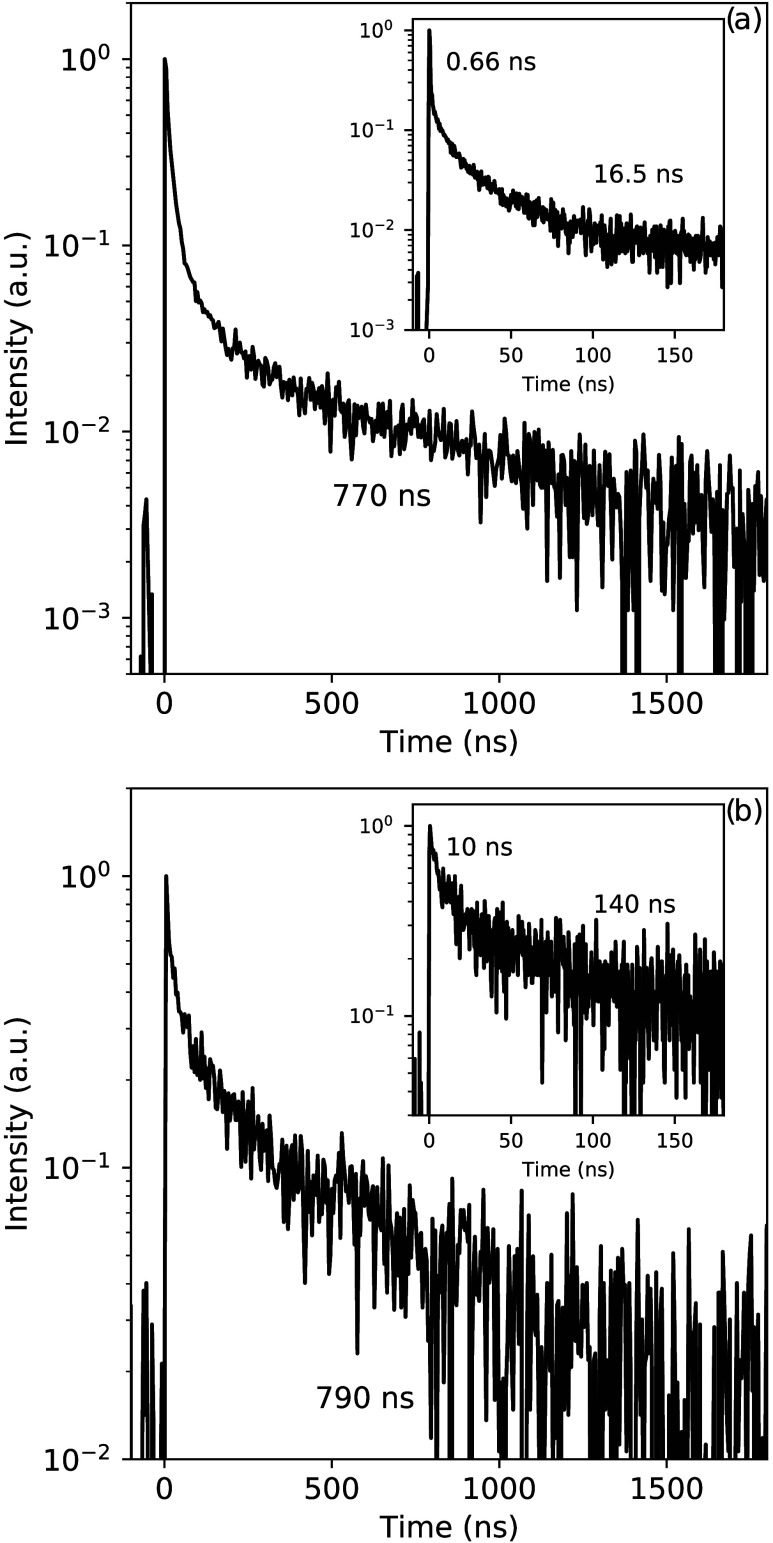
(a) Pulsed X-ray excited decay curve of the total emission spectrum of a Cs_4_PbBr_6_ single crystal with CsPbBr_3_ inclusions at 10 K. (b) Pulsed X-ray excited decay curve, recorded by placing a 630 nm long-pass filter in front of the detector, of a Cs_4_PbBr_6_ single crystal with CsPbBr_3_ inclusions at 10 K.

The 10 K pulsed X-ray excited decay curve of the total emission spectrum of the Cs_4_PbBr_6_ single crystal is shown in Figure S2a. The inset shows the first 150 ns of the decay curve, where two decay components of 0.66 and 16.5 ns can be observed. On longer time scales, a significantly slower component of 770 ns can also be observed. The measurement was repeated by placing a 630 nm long-pass filter in front of the photodetector, removing the contribution of both the 380 and 540 nm emissions. The resulting decay curve is shown in Figure 4b, with the inset showing the first 150 ns of the decay. The decay curve no longer contains the sub-nanosecond decay component observed in Figure 4a. The decay contains three decay components of 10, 140, and 790 ns. Nikl et al. have studied the decay behavior of Cs_4_PbBr_6_ with CsPbBr_3_ inclusions under photoexcitation finding a sub-nanosecond decay component for the 540 nm emission.^30^ Additionally the sub-nanosecond decay component is similar to the decay curves measured on CsPbBr_3_ single crystals under pulsed X-ray excitation.^3^ This suggests that the sub-nanosecond decay component observed in Figure 3a can be assigned to the 540 nm emission.

**Figure 5:**
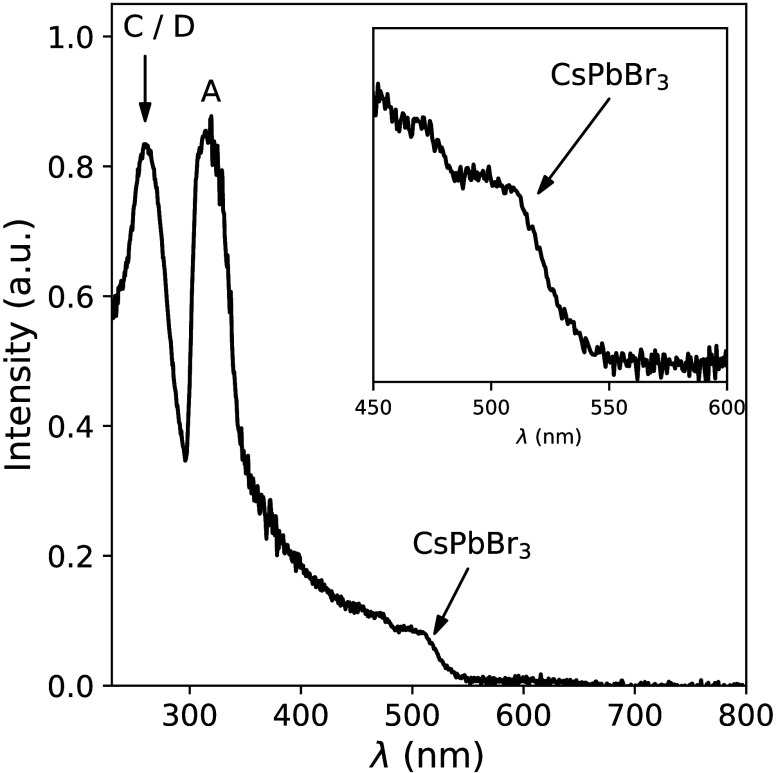
Room-temperature absorbance spectrum of a Cs_4_PbBr_6_ single crystal with CsPbBr_3_ inclusions; the inset shows a zoom-in of the feature observed between 500 and 550 nm.

Figure 5 shows the room-temperature absorbance spectrum of the Cs_4_PbBr_6_ single crystal, revealing two peaks at 260 nm (4.77 eV) and 315 nm (3.94 eV). An additional absorption edge is observed around 520 nm (2.38 eV). The 315 nm absorption band can be ascribed to the A-band transitions of Pb^2+^ (arrow 1 in Figure 2). Kondo et al. ascribed the 260 nm band to a combination of the C- and D-band transitions.^29^ The overlap of these two transitions has also been observed in alkali halides doped with low concentrations of Pb^2+^ for example KBr which behaves similarly to Cs_4_PbBr_6_.^77,78^ Hence, the 260 nm absorption band will be labeled as C/D-band transition (arrow 5 in Figure 2). The observed absorption edge, in Figure 5, around 520 nm (arrow 8 in Figure 2) can be ascribed to the presence of CsPbBr_3_ inclusions.^29,30^ For comparison, the absorption spectrum of a CsPbBr_3_ single crystal is shown in Figure S2, revealing a similar absorption edge around 520 nm. Based on these similarities and the powder diffraction pattern of the Cs_4_PbBr_6_ single crystals, the 540 nm emission is ascribed to CsPbBr_3_ inclusions (arrow 4 in Figure 2).

**Figure 6:**
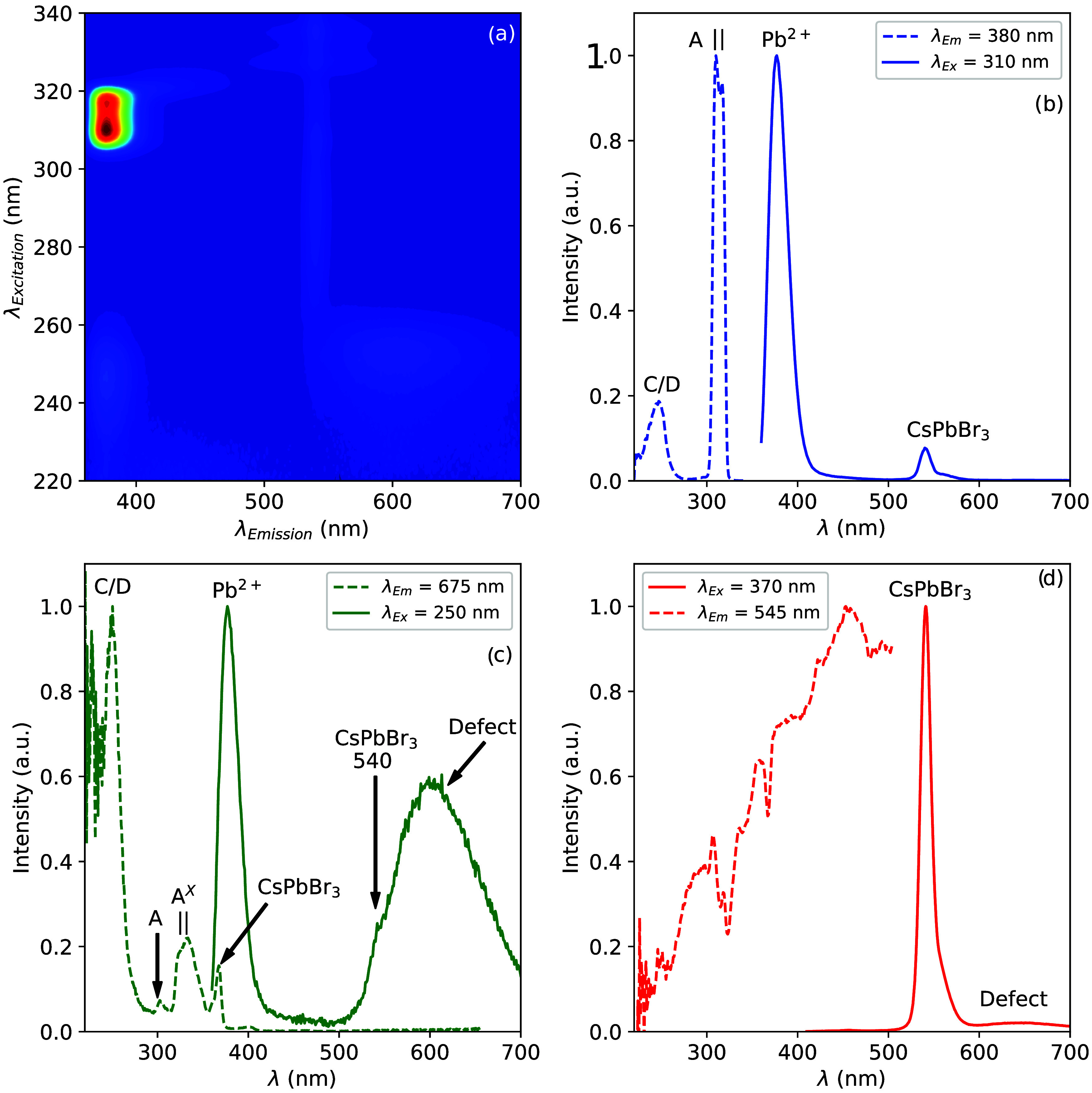
(a) Photoluminescence emission intensity of a Cs_4_PbBr_6_ single crystal with CsPbBr_3_ inclusions as a function of excitation wavelength at 10 K. Photoluminescence excitation and emission spectra at 10 K measured at emission and excitation wavelengths of (b) 380 and 310 nm, (c) 675 and 250 nm, and (d) 545 and 370 nm, respectively.

To further investigate the nature of the different absorption and emission bands, the photoluminescence emission of Cs_4_PbBr_6_ was measured as a function of the excitation wavelength at 10 K. The resulting image plot is shown in Figure 6a. From this image plot, two emission spectra are extracted at excitation wavelengths of 310 and 250 nm, as shown in Figure 6b,c, respectively. Upon excitation of the A-band (arrow 1 in Figure 2) at 310 nm, as shown in Figure 6b, two emission bands are observed at 378 and 540 nm. The 378 nm emission can be assigned to the intrinsic A-band emission of Cs_4_PbBr_6_ (arrow 2 in Figure 2),^30^ and the 540 nm emission to the presence of CsPbBr_3_ inclusions (arrow 4 in Figure 2).^19,29,30^ Analogous behavior is observed in Cs_4_PbCl_6_ due to the presence CsPbCl_3_ inclusions.^79^ The excitation spectrum of the 378 nm A-band emission in Figure 6b shows two excitation bands at 250 and 310 nm. Similar to the absorbance spectrum shown in Figure 5, these are assigned to the C/D- and A-band transitions (arrows 1 and 5 in Figure 2), respectively. Note that the 310 nm excitation band actually consists of two bands, at 310 and 317 nm, which is typical of the A-band and caused by a Jahn–Teller splitting.^34,35^ From the image plot in Figure 6a, it can be observed that the 378 nm emission band excited via the A band transition is the most intense emission band observed.

Upon excitation in the C/D-band (arrow 5 in Figure 2) at 250 nm, as shown in Figure 6c, two main emission bands at 378 and 610 nm are observed (arrows 2 and 6 in Figure 2). The 540 nm band is also present in the form of a shoulder on the shorter-wavelength side of the 610 nm band. The 610 nm emission band has not been observed before under photoexcitation but is the dominant emission band under X-ray excitation, as shown in Figure 3a. The excitation spectrum, recorded at 675 nm, in Figure 6c shows an intense 250 nm C/D- and a weak 310 nm A-band, which are also observed in Figure 6b but with a much different intensity ratio. Additionally, a second Jahn–Teller split A-band, (arrow 7 in Figure 2) is observed at 325 and 332 nm which is labeled as A^*X*^ and there is also an excitation band at 370 nm, attributed to the CsPbBr_3_ inclusions.^34,35^ Upon excitation at 370 nm (arrow 9 in Figure 2), two emission bands are observed at 540 and 610 nm, as shown in Figure 6d. The excitation spectrum recorded at 545 nm is also shown in Figure 6d.

The quenching behavior under optical excitation is similar to the quenching behavior under X-ray excitation. The *T*_50_ values for the 378, 540, and 600 nm emissions are approximately 110, 60, and 160 K. The temperature dependence of the different emission bands upon exciting at the 250 nm C/D-band, 310 nm A-band, and 370 nm CsPbBr_3_ inclusions is shown in Figure S3.

### III.II Cs_4_PbBr_6_ without CsPbBr_3_ Inclusions

Cs_4_PbBr_6_ without CsPbBr_3_ inclusions was synthesized using a solid-state synthesis. After the heat treatment of the CsBr:PbBr_2_ (4:1) mixture, a light gray powder was obtained. The X-ray diffraction pattern of the synthesized material only exhibits diffraction peaks of Cs_4_PbBr_6_ and CsBr. The diffraction pattern is shown in Figures S4 and S5 together with a two-phase Rietveld profile refinement. The refinement yielded expected mass fractions of >99% for Cs_4_PbBr_6_ and <1% for CsBr. In contrast to the diffraction pattern of the Cs_4_PbBr_6_ single crystal, no peaks related to CsPbBr_3_ were observed.

**Figure 7:**
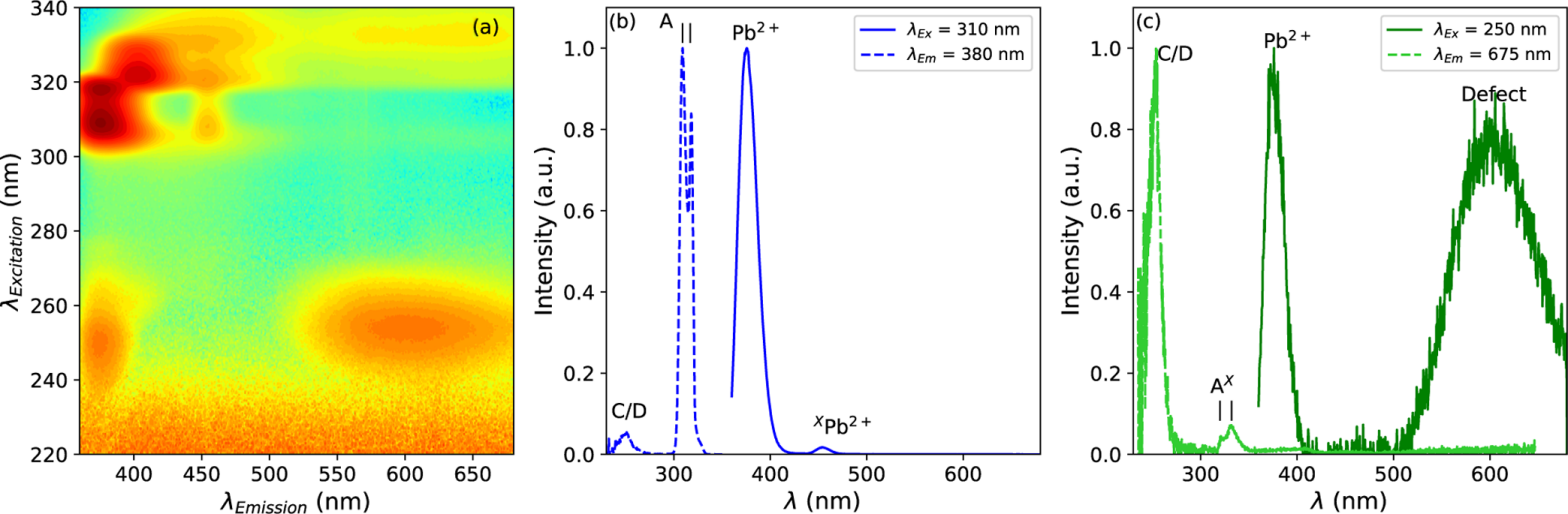
(a) Photoluminescence emission intensity of Cs_4_PbBr_6_ without CsPbBr_3_ inclusions as a function of excitation wavelength at 10 K, on a log scale. Photoluminescence excitation and emission spectra at 10 K measured at emission and excitation wavelengths of (b) 380 and 310 nm and (c) 675 and 250 nm, respectively.

To investigate the nature of the different absorption and emission bands of Cs_4_PbBr_6_ without CsPbBr_3_ inclusions, the photoluminescence emission was measured as a function of the excitation wavelength at 10 K. The resulting image plot, plotted on a log scale, is shown in Figure 7a. From the image plot, two emission spectra are extracted at excitation wavelengths of the 310 nm A-band and 250 nm C/D-band, shown in Figure 7b,c, respectively. Upon excitation of the A-band (arrow 1 in Figure 2), two emission bands at 378 and 455 (arrows 2 and 3 in Figure 2) are observed. The 540 nm emission (arrow 4 in Figure 2), observed in Figure 6b and related to the CsPbBr_3_ inclusions, is no longer present. The excitation spectrum of the 378 nm A-band Pb^2+^ emission, in Figure 7b, shows the C/D-band at 250 nm and the strong Jahn–Teller split A-band at 310 and 317 nm.^34,35^ Upon excitation of the C/D band (arrow 5 in Figure 2) both the 378 nm A-band emission and 610 nm emission (arrows 2 and 6 in Figure 2) are observed. The excitation spectrum recorded at 675 nm, in Figure 7c, contains the C/D band at 250 nm and the Jahn–Teller split A^*X*^-band at 320 and 332 nm.

**Figure 8:**
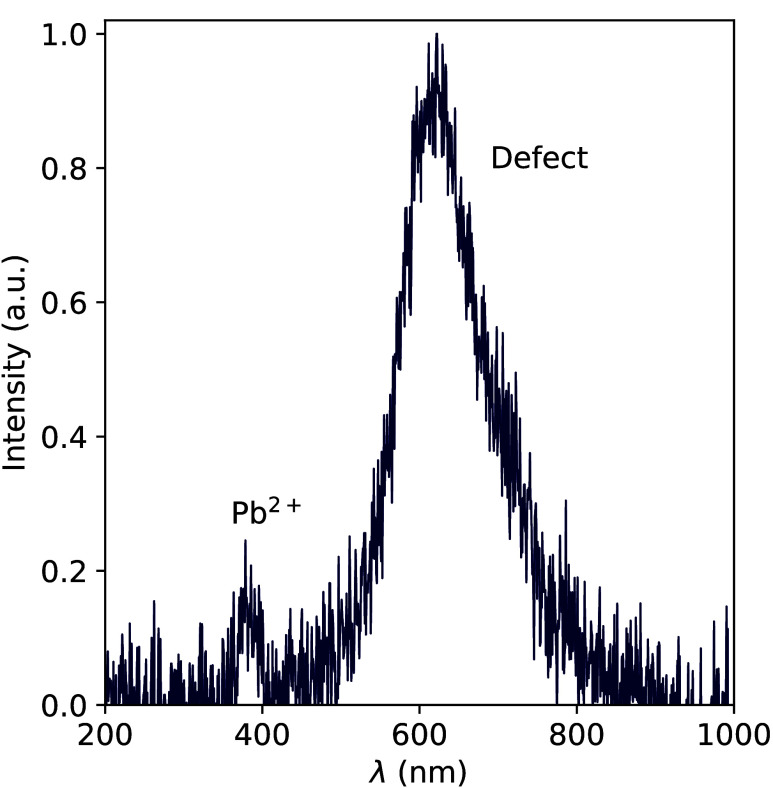
X-ray excited emission spectrum of Cs_4_PbBr_6_ without CsPbBr_3_ inclusions recorded at 10 K.

Figure 8 shows the 10 K X-ray excited emission spectrum of Cs_4_PbBr_6_ without CsPbBr_3_ inclusions. Two emission bands are observed at 380 and 610 nm with relative intensities of 1 and 25, respectively. The *T*_50_ temperature for both emission bands is 150 K as was obtained from temperature-dependent behavior of both bands, as shown in Figure S6a,b.

## IV Discussion

### IV.I Perturbed Pb^2+^ Coordination Sphere

**Figure 9:**
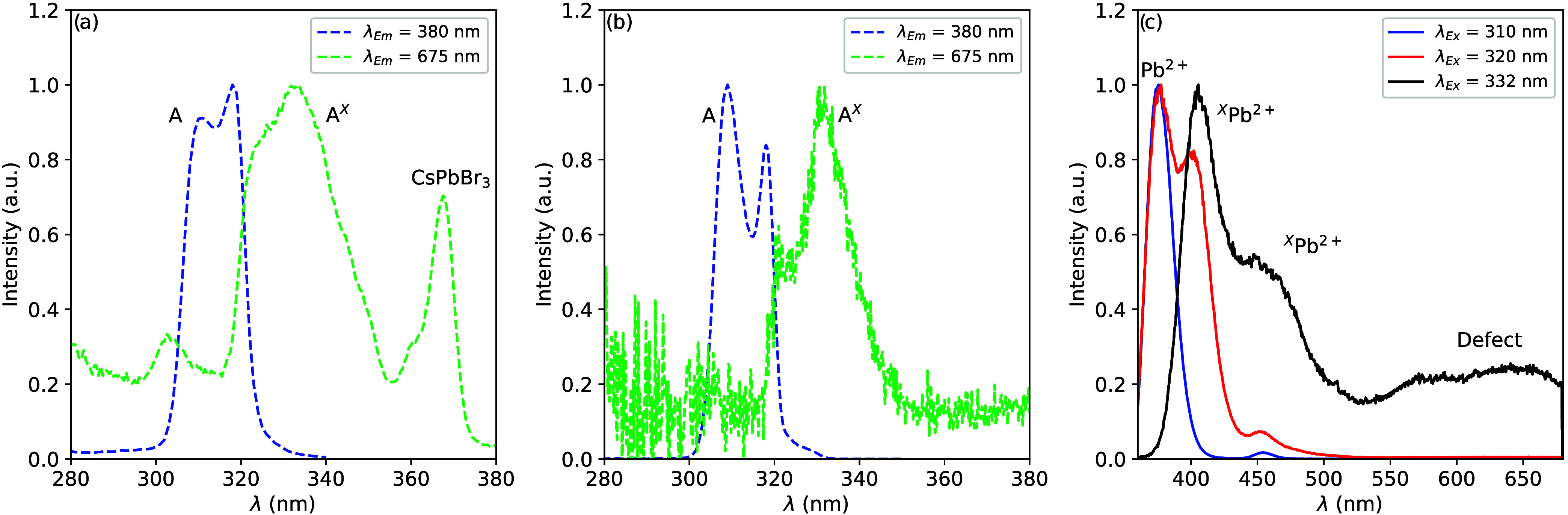
Comparison of the A-band transitions of (a) Cs_4_PbBr_6_ with CsPbBr_3_ inclusions and (b) Cs_4_PbBr_6_ without CsPbBr_3_ inclusions. The two excitation spectra shown for both forms of Cs_4_PbBr_6_ are recorded at 380 and 675 nm. The A-band transition of Pb^2+^ ions with an unperturbed coordination sphere is labeled A and that of Pb^2+^ ions with a perturbed coordination sphere is labeled A^*X*^. (c) Emission spectra of Cs_4_PbBr_6_ without CsPbBr_3_ inclusions excited in the A- and A^*X*^-bands.

Depending on the emission wavelength at which the excitation spectra are recorded, two different A-excitation bands are observed. A comparison of the two excitation bands for Cs_4_PbBr_6_ with and without CsPbBr_3_ inclusions is shown in Figure 9a,b, respectively. Figure 9c shows three emission spectra excited at 310 nm (only A-band transition, arrow 2 in Figure 2), 320 nm (mixed A- and A^*X*^-band transitions), and 332 nm (only A^*X*^-band transition, arrow 7 in Figure 2) of the Cs_4_PbBr_6_ sample without CsPbBr_3_ inclusions. Exciting only in the A-band yields two emission bands at 378 nm (strong) and 455 nm (weak). Exciting in the A^*X*^-band yields three emission bands at 405, 455, and 610 nm. It is known that the presence of defects close to Pb^2+^ ions influences both the excitation and emission properties of Pb^2+^ ions.^80^ This has been studied extensively in the alkali halides.^81−88^ Based on these observations, the A-band excitation and A-band emission (arrows 1 and 2 in Figure 2, respectively) are attributed to originate from a Pb^2+^ ion on an unperturbed site. The A^*X*^-band (arrow 7 in Figure 2) and the 405 and 455 nm emission bands (arrow 3 in Figure 2) originate from a Pb^2+^ ion with a different or perturbed coordination shell. The presence of two additional emission bands points to at least two different perturbed coordination spheres of Pb^2+^. These could, for example, be a Pb^2+^ interstitial, a bromine vacancy next to Pb^2+^, or Pb^2+^ on a Cs^+^ site.

After exciting the A-band transition, next to A-band emission, energy transfer to a Pb^2+^ ion with a perturbed coordination sphere can take place, as evident from the 455 nm emission band in Figure 7b. Energy transfer can also take place to the CsPbBr_3_ inclusions, if present, as evident from the 540 nm emission band in Figure 6b. After exciting the A^*X*^-band, next to the 405 and 455 nm emissions, the 610 nm emission band can also be observed, as seen in Figure 9c.

### IV.II Defect-Related Emission

The 610 nm emission band is mainly observed upon exiting the C/D-band at 250 nm, as evident from the intensities of the excitation bands shown in Figures 6c and 7c. It is also possible to observe the 610 nm emission upon exciting the A^*X*^-band, excluding D-band emission^89−91^ as potential explanation. The 610 nm emission excitation via the A-band is weak, excitation via the A^*X*^-band is significantly stronger as shown in Figure 6c. This all suggests that the 610 nm emission originates from a defect in the vicinity of a Pb^2+^ ion, resulting in a perturbation of the coordination sphere of Pb^2+^ and the observation of the A^*X*^-band. The 610 nm emission band cannot be excited directly. The defect seems to first need to trap a mobile charge carrier before it can become emissive. This can happen by exciting the C/D-band, upon which mobile charge carriers are created, or via excitation of a CsPbBr_3_ inclusion well above the bandgap, at 370 nm (arrow 9 in Figure 2), or a Pb^2+^ ion with a perturbed coordination sphere, illustrated by energy transfer pathways d, h, and f in Figure 2, respectively. The excitation spectra of the 610 nm emission, Figures 6c and 7c, show that exciting the C/D band is the dominant pathway to excite the defects.

### IV.III Inclusion-Related Emission

The emission peak, linked to the CsPbBr_3_ inclusions, was located at 540 nm in the Cs_4_PbBr_6_ single crystals used in this work. The green CsPbBr_3_ inclusion emission in nanocrystals however is typically reported to be located from 515 to 524 nm.^52^ It has been demonstrated by Chen et al. and Almeida et al., in Cs_4_PbBr_6_ nanocrystals with CsPbBr_3_ inclusions, that the emission wavelength of the CsPbBr_3_ emission can be tuned by altering the synthesis and effectively changing the size of the CsPbBr_3_ inclusions.^45,92^ The 540 nm emission wavelength observed in this work matches with the emission wavelength observed for bulk CsPbBr_3_.^3^ This suggests that during the synthesis process from the melt CsPbBr_3_ domains are formed large enough to not be influenced by confinement effects and show bulk properties. When Cs_4_PbBr_6_ with CsPbBr_3_ inclusions is excited at wavelengths longer than 350 nm it is possible to directly excite the CsPbBr_3_ inclusions, as demonstrated in Figure 6d. The inclusions can also be excited by an energy transfer process from either the A-band, C/D-band, or a Pb^2+^ ion with a perturbed coordination sphere, illustrated by energy transfer pathways b, e, and g in Figure 2, respectively.

### IV.IV Excitation and Emission Mechanism

Based on the results and discussion, a vacuum referred binding energy diagram can be constructed to explain the observed emission processes. The resulting diagram is shown in Figure 2. Based on computational methods presented by Kang et al.^68^ and information from He et al.^93^ we tentatively placed the valence band (VB) top of Cs_4_PbBr_6_ at −7 eV and that of CsPbBr_3_ 1 eV higher. The optical bandgap of Cs_4_PbBr_6_, which equals the A-band transition energy, is 3.9 eV. This value has been determined experimentally.^29,94^ The bottom of the conduction band (CB) of Cs_4_PbBr_6_ is determined by adding the optical bandgap to the VRBE of the top of the VB. The CB-bottom of CsPbBr_3_ is determined by adding the emission energy to the VRBE of its VB-top; due to the small Stokes shift of CsPbBr_3_, it can be assumed that the emission energy is approximately equal to the optical bandgap. It is estimated that the error margins in placing the top of the VB are approximately ±0.3 eV; essential is that the VB-top and CB-bottom of CsPbBr_3_ fall both well inside the bandgap of Cs_4_PbBr_6_. The C/D-band transition energy is defined as the mobility bandgap, where mobile charge carriers are created upon excitation. The position of the A- and C/D-band transitions is determined based on the excitation spectra shown in Figures 6b and 7b.

The diagram consists of two sections: on the left, the processes taking place in Cs_4_PbBr_6_. On the right, the additional processes are taking place due to the presence of CsPbBr_3_ inclusions. The position of the A^*X*^-band transitions, labeled ^*X*^Pb^2+^ are determined using Figures 6c and 7c. The exact position of the defect-related energy levels, labeled *X*_def_ in the diagram, is uncertain. The spacing between the levels is based of the emission wavelength of the defect-related emission.

After exciting the A-band (arrow 1), three processes can take place: A-band emission (arrow 2), or energy transfer (pathways a and b) resulting in emission from a ^*X*^Pb^2+^ ion (arrow 3) or CsPbBr_3_ inclusion (arrow 4). After exciting the C/D-band (arrow 5), three different processes can take place: A-band emission (arrow 2), or energy and/or charge carrier transfer (pathways d and e), resulting in emission from a defect (arrow 6) or CsPbBr_3_ inclusions (arrow 4). It is also possible to excite a ^*X*^Pb^2+^ ion (arrow 7) after which three processes can take place: A^*X*^-band emission (arrow 3) or energy transfer (pathways f and g) resulting in emission from a defect (arrow 6) or CsPbBr_3_ inclusions (arrow 4). The CsPbBr_3_ inclusions can also be excited directly (arrows 8 and 9) after which two processes can take place: emission from the inclusion (arrow 4) or energy transfer (pathway h) if excited well above the bandgap (arrow 9), resulting in emission from a defect (arrow 6).

### IV.V X-ray Versus Optical Excitation

Under X-ray excitation, as shown in Figure 3a, the 610 nm emission band is 40.6 times more intense compared with the A-band emission. Upon exciting the C/D-band the intensity of the 610 nm emission is only 2.5 times stronger compared to the A-band emission. Moreover, it is completely absent upon exciting the A-band. The observed intensity difference can be explained based on the difference between X-ray excitation and optical excitation. Upon X-ray excitation, all energy of the incident X-ray is transferred to a primary electron. This hot electron creates secondary excitations via electron–electron interactions along its ionization track. This results in the formation of spatially separated electrons and holes. The electrons, due to their larger mobility, will diffuse further away from the initial ionization track compared to the holes.^95−98^ The separated electrons and holes first need to approach each other before emission can take place. Upon optically exciting the C/D-band transition, the formed electrons and holes stay in closer proximity; emission can take place immediately. This happens on a shorter time scale compared to the recombination of the spatially separated electrons and holes formed upon X-ray excitation. The latter thus have more time to move through the crystal lattice and find a defect, thus leading to a more intense 610 nm emission band upon X-ray excitation.

## V Conclusions

In this work, the zero-dimensional cesium lead halide compound Cs_4_PbBr_6_ has been studied as a function of temperature under both X-ray and UV–vis excitation. Cs_4_PbBr_6_ is studied with and without CsPbBr_3_ inclusions. Upon X-ray excitation, most of the scintillation light is present in the 610 nm emission band for both forms of Cs_4_PbBr_6_. This is ascribed to the formation of spatially separated charge carriers reaching lattice defects. The pulsed X-ray excited decay curves show multiple decay components, the longest being approximately 800 ns. Upon exciting the C/D-band at 250 nm, the 610 nm emission band was also found which has not been reported before. The excitation spectrum of the 610 nm emission band contains an excitation band, labeled A^*X*^, which is ascribed to Pb^2+^ ions with a perturbed coordination sphere. It is suggested that the 610 nm emission originates from defects in the vicinity of Pb^2+^ ions, leading to the contraction of the coordination sphere. The 540 nm emission, based on the X-ray diffraction patterns and absorption spectra, is assigned to the presence of CsPbBr_3_ inclusions. It is thus shown experimentally that both CsPbBr_3_ inclusions and defects play a role in the photoluminescence behavior of Cs_4_PbBr_6_. All experimental results could be combined to formulate a general mechanism for the emission behavior of Cs_4_PbBr_6_.

## Supporting Information Available

Photoluminescence and scintillation mechanism of Cs$_4$PbBr$_6$ (ZIP)
X-ray diffraction pattern of Cs_4_PbBr_6_ grown using the vertical Bridgman method; absorbance spectrum of a CsPbBr_3_ single crystal; temperature-dependent photoluminescence emission spectra and integrated intensities of Cs_4_PbBr_6_ single crystal upon exciting at 250, 310, and 370 nm; X-ray diffraction pattern of synthesized Cs_4_PbBr_6_ powder; and temperature-dependent X-ray excited emission spectra of synthesized Cs_4_PbBr_6_ powder (PDF)

## References

[ref1] Bitowosuto M. D.; Cortecchia D.; Drozdowski W.; Brylew K.; Lachmanski W.; Bruno A.; Soci C. X-ray Scintillation in Lead Halide Perovskite Crystals. Sci. Rep. 2016, 6, 37254 10.1038/srep37254. 27849019 PMC5111063

[ref2] Li Y.; Shao W.; Ouyang X.; Zhu Z.; Zhang H.; Ouyang X.; Liu B.; Xu Q. Scintillation Properties of Perovskite Single Crystals. J. Phys. Chem. C 2019, 123 (28), 17449–17453. 10.1021/acs.jpcc.9b05269.

[ref3] van Blaaderen J. J.; Biner D.; Kramer K. W.; Dorenbos P. The Temperature Dependent Optical and Scintillation Characterisation of Bridgman Grown CsPbX_3_ (X = Br, Cl) Single Crystals, Nuclear Instruments. Nucl. Instrum. Methods Phys. Res., Sect. A 2024, 1064, 169322 10.1016/j.nima.2024.169322.

[ref4] van Blaaderen J. J.; Maddalena F.; Dong C.; Birowosuto M. D.; Dorenbos P. Temperature Dependent Scintillation Properties and Mechanisms of (PEA)_2_PbBr_4_ Single Crystals. J. Mater. Chem. C 2022, 10, 11598–11606. 10.1039/D2TC01483A. PMC9386685 36090966

[ref5] van Blaaderen J. J.; van der Sar S.; Onggo D.; K Sheikh M. A.; Schaart D. R.; Birowosuto M. D.; Dorenbos P. (BZA)_2_PbBr_4_: A Potential Scintillator for Photon-Counting Computed Tomography Detectors. J. Lumin. 2023, 263, 120012 10.1016/j.jlumin.2023.120012.

[ref6] van Blaaderen J. J.; van den Brekel L. A.; Kramer K. W.; Dorenbos P. Scintillation and Optical Characterisation of CsCu_2_I_3_ Single Crystals from 10 to 400K. Chem. Mater. 2023, 35, 9623–9631. 10.1021/acs.chemmater.3c01810. 38047185 PMC10687859

[ref7] Akkerman Q. A.; Manna L. What Defines a Halide Perovskite?. ACS Energy Lett. 2020, 5, 604–610. 10.1021/acsenergylett.0c00039. 33344766 PMC7739487

[ref8] Dorenbos P. The Quest for High Resolution γ-Ray Scintillators. Opt. Mater.: X 2019, 1, 100021 10.1016/j.omx.2019.100021.

[ref9] Dorenbos P. Fundamental Limitations in the Performance of Ce^3+^-, Pr^3+^-, and Eu^2+^- Activated Scintillators. IEEE Trans. Nucl. Sci. 2010, 57 (3), 1162–1167. 10.1109/TNS.2009.2031140.

[ref10] van Loef E. V. D.; Dorenbos P.; van Eijk C. W. E.; et al. High-Energy-Resolution Scintillator: Ce^3+^ activated LaBr_3_. Appl. Phys. Lett. 2001, 79, 1573 10.1063/1.1385342.

[ref11] Alekhin M. S.; Biner D. A.; Krämer K. W.; Dorenbos P. Optical and Scintillation Properties of CsBa_2_I_5_:Eu^2+^. J. Lumin. 2014, 145, 723–728. 10.1016/j.jlumin.2013.08.058.

[ref12] Bourret-Courchesne E. D.; Bizarri G.; Borade R.; Yan Z.; Hanrahan S. M.; Gundiah G.; Choudhry A.; Canning A.; Derenzo S. E. Eu^2+^-Doped CsBa_2_I_5_, a New High-Performance Scintillator. Nucl. Instrum. Methods Phys. Res. A 2009, 612, 138–142. 10.1016/j.nima.2009.10.146.

[ref13] van Aarle C.; Krämer K. W.; Dorenbos P. Avoiding Concentration Quenching and Self-Absorption in Cs_4_EuX_6_ (X = Br, I) by Sm^2+^ Doping. J. Mater. Chem. C 2023, 11, 2336–2344. 10.1039/D2TC05311J. PMC9909777 36777479

[ref14] van Aarle C.; Krämer K. W.; Dorenbos P. Characterisation of Sm^2+^-Doped CsYbBr_3_, CsYbI_3_ and YbCl_2_ for Near-Infrared Scintillator Application. J. Lumin. 2022, 251, 119209 10.1016/j.jlumin.2022.119209.

[ref15] Wolszczak W. W.; Carrroll D. L.; Williams R. T. Advanced X-ray Detector Technologies, Chapter 1Springer, 2022.

[ref16] Williams R. T.; Wolszczak W. W.; Yan X.; Carrol D. L. Perovskite Quantum-Dot-in-Host for Detection of Ionising Radiation. ACS Nano 2020, 14, 5161–5169. 10.1021/acsnano.0c02529. 32401004

[ref17] Saki Z.; Byranvand M. M.; Taghavinia N.; Kedia M.; Saliba M. Solution-Processed Perovskite Thin-Films: The Journey from Lab- to Large-Scale Solar Cells. Energy Environ. Sci. 2021, 14, 5690–5722. 10.1039/D1EE02018H.

[ref18] Dunlap-Shohl W. A.; Zhou Y.; Padture N. P.; Mitzi D. B. Synthetic Approaches for Halide Perovskite Thin Films. Chem. Rev. 2019, 119, 3193–3295. 10.1021/acs.chemrev.8b00318. 30387358

[ref19] Akkerman Q. A.; Raino G.; Kovalenko M. V.; Manna L. Genesis, Challenges and Opportunities for Colloidal Lead Halide Perovskite Nanocrystals. Nat. Mater. 2018, 17, 394–405. 10.1038/s41563-018-0018-4. 29459748

[ref20] Song K. S.; Williams R. T. Springers Series in Solid State Sciences, Self traped Excitons, 2nd ed.; SpringerVerlag: Berlin, 1996; p 105.

[ref21] Yuan Z.; Zhou C.; Tian Y.; Shu Y.; Messier J.; Wang J. C.; van de Burght L. J.; Kountouriotis Y.; Xin E.; Holt E.; et al. One-Dimensional Organic Lead Halide Perovskite with Efficient Bluish White-Light Emission. Nat. Commun. 2017, 8, 14051 10.1038/ncomms14051. 28051092 PMC5216108

[ref22] Dohner E. R.; Jaffe A.; Bradshaw L. R.; Karunadasa H. I. Intrinsic White-Light Emission from Layered Hybrid Perovskites. J. Am. Chem. Soc. 2014, 136 (38), 13154–13157. 10.1021/ja507086b. 25162937

[ref23] Mao L.; Wy Y.; Stoumpos C. C.; Wasielewski M. R.; Kanatzidis M. G. White-Light Emission and Structural Distortion in New Corrugated Two-Dimensinal Lead Bromide Perovskties. J. Am. Chem. Soc. 2017, 139 (14), 5210–5215. 10.1021/jacs.7b01312. 28306254

[ref24] Maddalena F.; Xie A.; Arramel; Witkowski M. E.; Makowski M.; Mahler B.; Drozdowski W.; Mariyappan T.; Springham S. V.; Coquet P.; et al. Effect of Commensurate Lithium Doping on the Scintillation of Two-Dimensional Perovskite Crystals. J. Mater. Chem. C 2021, 9, 2504 10.1039/D0TC05647B.

[ref25] Xie A.; Maddalena F.; Witkowski M. E.; Makowski M.; Mahler B.; Drozdowski W.; Springham S. V.; Coquet P.; Dujardin C.; Birowosuto M. D.; Dang C. Library of Two-Dimensional Hybrid Lead Halide Perovskite Scintillator Crystals. Chem. Mater. 2020, 32, 8530–8539. 10.1021/acs.chemmater.0c02789.

[ref26] MØller C. K. On the Structure of Cesium Hexahalogeno-Plumbates (II); Copenhagen: Denmark: Munksgaard, 1960; Vol. 32, pp 1–13.

[ref27] De Bastiani M.; Dursun I.; Zhang Y.; Alshankiti B. A.; Miao X.-H.; Yin J.; Yengel E.; Alarousu E.; Turedi B.; Almutlaq J. M.; et al. Inside Perovskites: Quantum Luminescence from Bulk Cs_4_PbBr_6_ Single Crystals. Chem. Mater. 2017, 29, 7108–7113. 10.1021/acs.chemmater.7b02415.

[ref28] Akkerman Q. A.; Park S.; Radicchi E.; Nunzi F.; Mosconi E.; De Angelis F.; Brescia R.; Rastogi P.; Prato M.; Manna L. Nearly Monodisperse Insulator Cs_4_PbX_6_ (X = Cl, Br, I) Nanocrystals, Their Mixed Halide Compositions, and Their Transformation into CsPbX_3_ Nanocrystals. Nano Lett. 2017, 17, 1924–1930. 10.1021/acs.nanolett.6b05262. 28196323 PMC5345893

[ref29] Kondo S.; Amaya K.; Saito T. Localised Optical Absorption in Cs_4_PbBr_6_. J. Phys.: Condens. Matter 2002, 14, 2093–2099. 10.1088/0953-8984/14/8/334.

[ref30] Nikl M.; Mihokova E.; Nitsch K.; Somma F.; Giampaolo C.; Pazzi G. P.; Fabeni P.; Zazubovich S. Photoluminescence of Cs_4_PbBr_6_ Crystals and Thin Films. Chem. Phys. Lett. 1999, 306, 5–6. 10.1016/S0009-2614(99)00477-7.

[ref31] Lin H.; Zhou C.; Tian Y.; Siegrist T.; Ma B. Low-Dimensional Organometal Halide Perovskites. ACS Energy Lett. 2018, 3 (1), 54–62. 10.1021/acsenergylett.7b00926.

[ref32] Saidaminov M. I.; Mohammed A. F.; Bakr A. M. Low-Dimensional-Networked Metal Halide Perovskites: The Next Big Thing. ACS Energy Lett. 2017, 2 (4), 889–896. 10.1021/acsenergylett.6b00705.

[ref33] Bohun A.; Dolejsi J.; Barta C. The Absorption and Luminescence of (PbCl_6_)^4–^ and (PbBr_6_)^4–^ Complexes. Czech. J. Phys. B 1970, 20, 803–807. 10.1007/BF01726608.

[ref34] Jacobs P. W. M. Alkali Halide Crystals Containing Impurity Ions with the Ns^2^ Ground-State Electronic Configuration. J. Phys. Chem. Solids 1991, 52 (1), 35–67. 10.1016/0022-3697(91)90059-9.

[ref35] Ranfagni A.; Mugnai D.; Bacci M.; et al. The Optical Properties of Thallium-Like Impurities in ALkali-Halide Crystals. Adv. Phys. 1983, 32 (6), 823–905. 10.1080/00018738300101621.

[ref36] Forro M. Uber die Absorptionspektra Einiger Alkalihalogenidphosphore Bei Hohen Temperaturen. Z. Phys. 1929, 56, 534–543. 10.1007/BF01338864.

[ref37] Blasse G. Optical Electron Transfer between Metal Ions and Its Consequences Springer: Berlin, Heidelberg pp 153–187.

[ref38] Folkerts H. F.; Hamstra M. A.; Blasse G. The Luminscence of Pb^2+^ in Alkaline Earth Sulfates. Chem. Phys. Lett. 1995, 246, 135–138. 10.1016/0009-2614(95)01067-J.

[ref39] Zhang Y.; Saidaminov M. I.; Dursun I.; Yang H.; Murali B.; Alarousu E.; Yengel E.; Alshankiti B. A.; Bakr A. M.; Mohammed A. F. Zero-Dimensional Cs_4_PbBr_6_ Perovskite Nanocrystals. J. Phys. Chem. Lett. 2017, 8 (5), 961–965. 10.1021/acs.jpclett.7b00105. 28181438

[ref40] Liu Z.; Bekenstein Y.; Ye X.; Nguyen S. N.; Swabeck J.; Zhang D.; Lee S. T.; Yang P.; Ma W.; Alivisatos A. P. Ligand Mediated Transformation of Cesium Lead Bromide Perovskite Nanocrystals to Lead Depleted Cs_4_PbBr_6_ Nanocrystals. J. Am. Chem. Soc. 2017, 139 (15), 5309–5312. 10.1021/jacs.7b01409. 28358191

[ref41] Yin J.; Zhang Y.; Bruno A.; Soci C.; Bakr A. M.; Bredas J. L.; Mohammad A. F. Intrinsic Lead ion Emission in Zero-Dimensional Cs_4_PbBr_6_ Nanocrystals. ACS Energy Lett. 2017, 2 (12), 2805–2811. 10.1021/acsenergylett.7b01026.

[ref42] Wang X.; Liu Y.; Liu N.; Sun R.; Zheng W.; Liu H.; Zhang Y. Revisiting the nanocrystal formation process of zero-dimensional perovskite. J. Mater. Chem. A 2021, 9, 4658–4663. 10.1039/D1TA00428J.

[ref43] Cai H.; Lao M.; Xu J.; Chen Y.; Zhong C.; Lu S.; Hao A.; Chen R. All-Inorganic Perovskite Cs_4_PbBr_6_ Thin Films in Optoelectronic Resisitve Switching Memory Devices with a Logical Application. Ceram. Int. 2019, 45 (5), 5724–5730. 10.1016/j.ceramint.2018.12.038.

[ref44] Cha J. H.; Han J. H.; Yin W.; Park C.; Park Y.; Ahn T. K.; Cho J. H.; Jung D. Y. Photoresponse of CsPbBr_3_ and Cs_4_PbBr_6_ Perovskite Single Crystals. J. Phys. Chem. Lett. 2017, 8 (3), 565–570. 10.1021/acs.jpclett.6b02763. 28067051

[ref45] Chen X.; Zhang F.; Ge Y.; Shi L.; Huang S.; Tang J.; Lv Z.; Zhang L.; Zou B.; Zhong H. Centimeter-Sized Cs_4_PbBr_6_ Crystals with Embedded CsPbBr_3_ Nanocrystals Showing Superior Photoluminescence: Nonstoichiometry Induced Transformation and Light-Emitting Applications. Adv. Funct. Mater. 2018, 28, 1706567 10.1002/adfm.201706567.

[ref46] Saidaminov M. I.; Almutlaq J.; Sarmah S.; Dursun I.; Zhumekenov A. A.; Begum R.; Pan J.; Cho N.; Mohammed A. F.; Bakr A. M. Pure Cs_4_PbBr_6_: Highly Luminescent Zero-Dimensional Perovskite Solids. ACS Energy Lett. 2016, 1 (4), 840–845. 10.1021/acsenergylett.6b00396.

[ref47] Zhou Y.; Ding J.; Wang Z.; Tong Y.; Liang X.; Du J.; Xia W.; Liu Z.; Xiang W. Ultrastable EVA Film-Protected Cs_4_PbBr_6_ Solid Powder for Wide Color Gamut Blacklight Display and Upconversion Emission. Chem. Eng. J. 2021, 426, 130786 10.1016/j.cej.2021.130786.

[ref48] Bao Z.; Wang H.-C.; Jiang Z.-F.; Chung R. J.; Liu R.-S. Continuous Synthesis of Highly Stable Cs_4_PbBr_6_ Perovskite Microcrystals by a Microfluidic System and Their Application in Whit-Light-Emitting Diodes. Inorg. Chem. 2018, 57 (21), 13071–13074. 10.1021/acs.inorgchem.8b01985. 30351076

[ref49] Chen Y.-M.; Zhou Y.; Zhao Q.; Zhang J.-Y.; Ma J.-P.; Xuan T.-T.; Guo S.-Q.; Yong Z.-J.; Wang J.; Kuroiwa Y.; et al. Cs_4_PbBr_6_/CsPbBr_3_ Perovskite Composites with Near-Unity Luminescence Quantum Yield: Large-Scale Synthesis, Luminescence and Formation Mechanism, and White Light Emitting Diode Application. ACS Appl. Mater. Interfaces 2018, 10 (18), 15905–15912. 10.1021/acsami.8b04556. 29668249

[ref50] Zhao H.; Sun R.; Wang Z.; Fu K.; U X.; Zhang Y. Zero-Dimensional Perovskite Nanocrystals for Efficient Luminescent Solar Concentrators. Adv. Funct. Mater. 2019, 29 (30), 1902262 10.1002/adfm.201902262.

[ref51] Liu Y.; Li N.; Sun R.; Zheng W.; Liu T.; Li H.; Chen Y.; Liu G.; Zhao H.; Liu H. Stable metal-halide perovskites for luminescent solar concentrators of real-device integration. Nano Energy 2021, 85, 105960 10.1016/j.nanoen.2021.105960.

[ref52] Akkerman Q. A.; Abdelhady A. L.; Manna L. Zero-Dimensinoal Cesium Lead Halides: History, Properties, and Challenges. J. Phys. Chem. Lett. 2018, 9 (9), 2326–2337. 10.1021/acs.jpclett.8b00572. 29652149 PMC5937914

[ref53] Cha J. H.; Lee H. J.; Kim S. H.; Ko K. C.; Suh B. J.; Han O. H.; Jung D. Y. Superparamagnetism of Green Emissive Cs_4_PbBr_6_ Zero-Dimensional Perovskite Crystals. ACS Energy Lett. 2020, 5 (7), 2208–2216. 10.1021/acsenergylett.0c00964.

[ref54] Wang K.; Yuan Y.; Du S.; Yao Q.; Zhang J.; Chang J.; Shang C.; Li C.; Sun H.; Zhang W.; Ding J. Understanding of the Photoluminescence Mechanism Based on Zero-DImensional Cs_4_PbBr_6–*m*_X_*m*_ (X = Cl, I) Single Crystals. J. Phys. Chem. C 2021, 125 (28), 15223–15232. 10.1021/acs.jpcc.1c04696.

[ref55] Seth S.; Samanta A. Fluorescent Phase-Pure Zero-Dimensional Perovskite Related Cs_4_PbBr_6_ Microdisks: Synthesis and Single-Particle Imaging Study. J. Phys. Chem. Lett. 2017, 8 (18), 4461–4467. 10.1021/acs.jpclett.7b02100. 28862458

[ref56] Qin Z.; Dai S.; Hadjiev V. G.; Wang C.; Xie L.; Ni Y.; Wu C.; Yang G.; Chen S.; Deng L.; et al. Revealing the Origin of Luminescence Center in 0D Cs4PbBr6 Perovskite. Chem. Mater. 2019, 31 (21), 9098–9104. 10.1021/acs.chemmater.9b03426.

[ref57] Zhang Z.; Zhu Y.; Wang W.; Zheng W.; Lin R.; Li X.; Zhang H.; Zhong D.; Huang F. Aqueous Solution Growth of Millimeter-Sized nongreen-Luminescent Wide Bandgap Cs_4_PbBr_6_ Bulk Crystal. Cryst. Growth Des. 2018, 18 (11), 6393–6398. 10.1021/acs.cgd.8b00817.

[ref58] Quan L. N.; Quintero-Bermudez R.; Voznyyy O.; Walters G.; Jain A.; Fan J. Z.; Zheng X.; Yang Z.; Sargent E. H. Highly Emissive Green Perovskite Nanocrystals in a Solid State Crystalline Matrix. Adv. Mater. 2017, 29 (21), 1605945 10.1002/adma.201605945. 28370565

[ref59] Ray A.; Maggioni D.; Baranov D.; Dang Z.; Prato M.; Akkerman Q. A.; Goldoni L.; Caneva E.; Manna L.; Abdelhady A. L. Green-Emitting Powders of Zero-Dimensional Cs_4_PbBr_6_: Delineating the Intricacies of the Synthesis and the Origin of Photoluminescence. Chem. Mater. 2019, 31 (18), 7761–7769. 10.1021/acs.chemmater.9b02944. 32952293 PMC7116092

[ref60] Riesen N.; Lockrey M.; Badek K.; Riesen H. On the Origins of the Green Luminscence in the ’”Zero-Dimensional Perovskite”Cs_4_PbBr_6_”: Conclusive Results from Cathodoluminescence Imaging. Nanoscale 2019, 11, 3925 10.1039/C8NR09255A. 30761398

[ref61] Wang L.; Liu H.; Zhang Y.; Mohammed A. F. Photoluminescence Origin of Zero-DImensional Cs_4_PbBr_6_ Perovskite. ACS Energy Lett. 2020, 5 (1), 87–99. 10.1021/acsenergylett.9b02275.

[ref62] Biswas K. Revisiting the Origin of Green Emission in Cs_4_PbBr_6_. Mater. Adv. 2022, 3 (17), 6791–6798. 10.1039/D2MA00544A.

[ref63] Cola M.; Massarotti V.; Riccardi R.; Sinistri C. Binary Systems Formed by Lead Bromide with (Li, Na, K, Rb, Cs, and Tl)Br: a DRA and Diffractometric Study. Z. Naturforsch., A 1971, 26.8, 1328–1332. 10.1515/zna-1971-0812.

[ref64] Nikl M.; Nitsch K.; Mikokova E.; Polak K.; Fabeni P.; Pazzi G. P.; Gurioli M.; Santucci S.; Phani R.; Scacco A.; et al. Lumienscence of CsPbBr_3_-Like Quantum Dots in CsBr Single Crystals. Phys. E: Low-Dimens. Syst. Nanostruct. 1999, 4 (4), 323–331. 10.1016/S1386-9477(99)00016-8.

[ref65] Aceves R.; Babin V.; Barboza Flores M.; Fabeni P.; Maaroos A.; Nikl M.; Nitsch K.; Pazzi G. P.; Perez Salas R.; Sildos I.; et al. Spectroscopy of CsPbBr_3_ Quantum Dots in CsBr:Pb Crystals. J. Lumin. 2001, 93 (1), 27–41. 10.1016/S0022-2313(01)00175-2.

[ref66] Nikl M.; Nitsch K.; Polak K.; Mihokova E.; Zazubovich S.; Pazzi G. P.; Fabeni P.; Salvini L.; Aceves R.; Barbose-Flores M.; et al. Quantum Size Effect in the Excitonic Luminescence of CsPbX_3_-Like Quantum Dots in CsX (X = Cl, Br) Single Crystal Host. J. Lumin. 1997, 72–74, 377–379. 10.1016/S0022-2313(96)00341-9.

[ref67] van Hattem A.; Alders D.; konings R. J. M.; Smith A. L. Ternary System CsI-PbI_2_-BiI_3_ and Thermodynamic Stability of Cesium Metal Halide Perovskites. J. Phys. Chem. C 2023, 127 (35), 17482–17496. 10.1021/acs.jpcc.3c02696.

[ref68] Kang B.; Biswas K. Exploring Polaronic, Excitonic Structures and Luminescence in Cs_4_PbBr_6_/CsPbBr_3_. J. Phys. Chem. Lett. 2018, 9 (4), 830–836. 10.1021/acs.jpclett.7b03333. 29390608

[ref69] Rietveld H. M. A Profile Refinement Method for Nuclear and Magnetic Structures. Appl. Crystallogr. 1969, 2, 64–71. 10.1107/S0021889869006558.

[ref70] van Laar B.; Schenk H. The Development of Powder Profile Refinement at the Reactor Centre Nehterlands at Petten. Acta Crystallogr., Sect. A: Found. Adv. 2018, 74.2, 88–92. 10.1107/S2053273317018435. 29493537 PMC5831587

[ref71] Rodríguez-Carvajal J. LLB Sacley and LCSIM Rennes. 2003.

[ref72] Roisnel T.; Rodríguez-Carvajal J. A Windows Tool for Powder Diffraction Patterns Analysis, Material Science Forum. In Proceedings of the European Powder Diffraction Conference (EPDIC7) 2000; pp 118–123.

[ref73] Kubota T.; Yanagimoto S.; Saito H.; Akiba K.; Ishii A.; Sannomiya T. Chatodoluminscence Spectral and Lifetime Mapping of Cs_4_PbBr_6_: Fast Lifetime and its Scintillator Application. Appl. Phys. Express 2024, 17, 015005 10.35848/1882-0786/ad1bc4.

[ref74] Ding Y.; Lin R.; Lioang Y.; Zheng W.; Chen L.; Ouyang X.; Huang F. High-Efficiency Down-Conversion Radiation Fluorescence and Ultrafast Photoluminscence (1.2 ns) at the Interface of Hybrid Cs_4_PbBr_6_ - CsI Nanocrystals. J. Phys. Chem. Lett. 2021, 12 (30), 7342–7349. 10.1021/acs.jpclett.1c01615. 34323502

[ref75] Li Y.; Chen L.; Liu B.; Ruan J.; Liu J.; Ouyang X.; Xu Q. The Phosphorescence Emission in Undoped Lead-Halide Cs_4_PbvBr_6_ Single Crystals at Low Temperature. Ceram. Int. 2022, 48 (12), 16730–16736. 10.1016/j.ceramint.2022.02.222.

[ref76] Wu X.; Zhou Q.; Wu H.; Du X.; Niu G.; Liang G.; Hy Q.; Xiao J. Cs_4_PbBr_6–*x*_Cl_*x*_ Single Crystals with Tunable Emission for X-ray Detection and Imaging. J. Phys. Chem. C 2021, 125, 26619–26626. 10.1021/acs.jpcc.1c08178.

[ref77] Collins P. R.; Fredericks W. J. Absorption Spectra and Oscillator Strength of KBr:Pb. J. Phys. Chem. Solids 1986, 47 (5), 529–532. 10.1016/0022-3697(86)90053-3.

[ref78] Sastry S. B. S.; Viswanathan V.; Ramasastry C. Lead Centres in Alkali Halides: NaCl, KCl and KBr. J. Phys. Soc. Jpn. 1973, 35, 508–513. 10.1143/JPSJ.35.508.

[ref79] Nikl M.; Mihokova E.; Nitsch K. Photoluminescence and Decay Kinetics of Cs_4_PbCl_6_ Single Crystals. Solid State Commun. 1992, 4 (12), 1089–1092. 10.1016/0038-1098(92)90691-2.

[ref80] Zazubovich S. Polarization Spectroscopy of ns^2^ Impurity Ions in Alkali Halides. Int. J. Modern Phys. B 1994, 08 (8), 985–1031. 10.1142/S0217979294000506.

[ref81] Bol A. A.; Meijerink A. Luminescence of Nanocrystaline ZnS:Pb^2+^. Phys. Chem. Chem. Phys. 2001, 3, 2105–2112. 10.1039/b100968k.

[ref82] Goovaerts E.; Nistor S. V.; Schoemaker D. Electron-Spin Resonance of a Complex Pb^+^ (6p^1^) Defectr in Alkali Halides. Phys. Rev. B 1983, 28, 3712 10.1103/PhysRevB.28.3712.

[ref83] Kerssen J.; De Gruijter W. G.; Volger J. EPR of Pb^+^ Ions and Exchange-Coupled Pb^+^ Ion Pairs in U.V.-Irradiated PbCl_2_ and PbBr_2_ Crystals. Physica 1973, 70 (2), 375–396. 10.1016/0031-8914(73)90255-3.

[ref84] Fabeni P.; Krasnikov A.; Nikl M.; Pazzi G. P.; Zazubovich S. Stimulated Self-Trapped Exciton Emission in CsI:Pb. Solid State Commun. 2003, 126 (12), 665–669. 10.1016/S0038-1098(03)00333-8.

[ref85] Egemberdiev Zh.; Nagirnyi V.; Soovik T.; Zazubovich S. Decay Kinetics and Polarization of the A_*T*_ Emission of Pb^2+^ Centres of Different STructure in KBr:PbBr_2_. Phys Status Solid (b) 1984, 126 (1), 407–414. 10.1002/pssb.2221260147.

[ref86] Egemberdiev Zh.; Usarov S.; Zazubovich S. Luminescene of Lead Ions Associated with Interstitials and Vacancies in Alkali Halides. Phys Status Solid (b) 1991, 164 (1), 195–206. 10.1002/pssb.2221640120.

[ref87] Egemberdiev Zh.; Ismailov K.; Usarov A.; Zazubovich S.; Jaanson N. Luminescent Associates of Pb^2+^ V_*c*_^–^ Dipoles with Interstitial Iodine Atoms in KI:PbI_2_ Crystals. Phys Status Solid (b) 1991, 163 (1), 183–190. 10.1002/pssb.2221630118.

[ref88] Nagli L. E.; Dyachenko S. V. Influence of a V_*c*_^–^ Vacancy on Luminescence of Pb^+^ Centres in Alkali Halides. Basic Solid State Phys. 1988, 146 (1), 295–301. 10.1002/pssb.2221460131.

[ref89] van Dijken A.; Folkerts H. F.; Blasse G. Evidence for D-band Emission from Pb^2+^ in Alkaline-Earth Fluorohalides with the PbFCl Struvture. J. Lumin. 1997, 72–74, 660–661. 10.1016/S0022-2313(96)00431-0.

[ref90] Folkerts H. F.; Ghianni F.; Blasse G. Search for D-level Emission of Pb^2+^ in Alkaline-Earth Aluminates and Gallates. J. Phys. Chem. Solids 1996, 57 (11), 1659–1665. 10.1016/0022-3697(96)00041-8.

[ref91] Folkerts H. F.; van Dijken A.; Blasse G. Two Types of Luminescence from Pb^2+^ in Alkaline-Earth Fluorohalides with the PbFCl Structure. J. Phys.: Condens. Matter 1995, 7 (50), 10049 10.1088/0953-8984/7/50/034.

[ref92] Almeida G.; Goldoni L.; Akkerman Q.; Dang Z.; Khan A. H.; Marras S.; Moreels I.; Manna L. Role of Acid-Base Eqwuilibira in the Size, Shape, and Phase Contril of Cesium Lead Bromide Nanocrystals. ACS Nano 2018, 12 (2), 1704–1711. 10.1021/acsnano.7b08357. 29381326 PMC5830690

[ref93] He Y.; Matei L.; Jung H. J.; McCall K. M.; Chen M.; Stoumpos C. C.; Liu Z.; Peters J. A.; Chung D. Y.; Wessels B. W.; et al. High Spectral Resolution of Gamma-Rays at Room Temperature by Perovskite CsPbBr_3_ Single Crystals. Nat. Commun. 2018, 9, 1609 10.1038/s41467-018-04073-3. 29686385 PMC5913317

[ref94] shin M.; Nam S. W.; Sadhanala A.; Shivanna R.; Anaya M.; Jimenez-Solano A.; Yoon H.; Jeon S.; Stranks S. D.; Hoye R. L. Z.; et al. Understanding the Origin of ultrasharp Sub-Bandgap Luminscence from Zero-Dimensional Inorganic Perovskite Cs_4_PbBr_6_. ACS Appl. Energy Mater. 2020, 3 (1), 192–199. 10.1021/acsaem.9b02214.

[ref95] Williams R. T.; Grim J. Q.; Li Q.; Ucer K. B.; Moses W. W. Excitation Density, Diffusion-Drift, and Proportionality in Scintillators. Phys Status Solid (b) 2011, 248, 426–438. 10.1002/pssb.201000610.

[ref96] Moses W. W.; Bizarri G.; Williams R. T.; Payne S. A.; Vasilev A. N.; Singh J.; Li Q.; Grim J. Q.; Choong W.-S. The Origins of Scintillator Non-Proportionality. IEEE Trans. Nucl. Sci. 2012, 59, 2038–2044. 10.1109/TNS.2012.2186463.

[ref97] Vasil’ev A. N. From Luminscence Non-Linearity to Scintillation Non-Proportionality. IEEE Trans. Nucl. Sci. 2008, 55 (3), 1054–1061. 10.1109/TNS.2007.914367.

[ref98] Khodyuk I. V. Nonproportionality of inorganic scintillators 10.4233/uuid:cb4008a8-981a-4283-b213-199d41756269.

