# Supplementary figures and images for: Photoluminescence and Scintillation Mechanism of Cs4PbBr6
Source: J Phys Chem C Nanomater Interfaces. 2024 Nov 12;128(46):19921–32. doi: 10.1021/acs.jpcc.4c06347 (PMC11587101; doi:10.1021/acs.jpcc.4c06347)

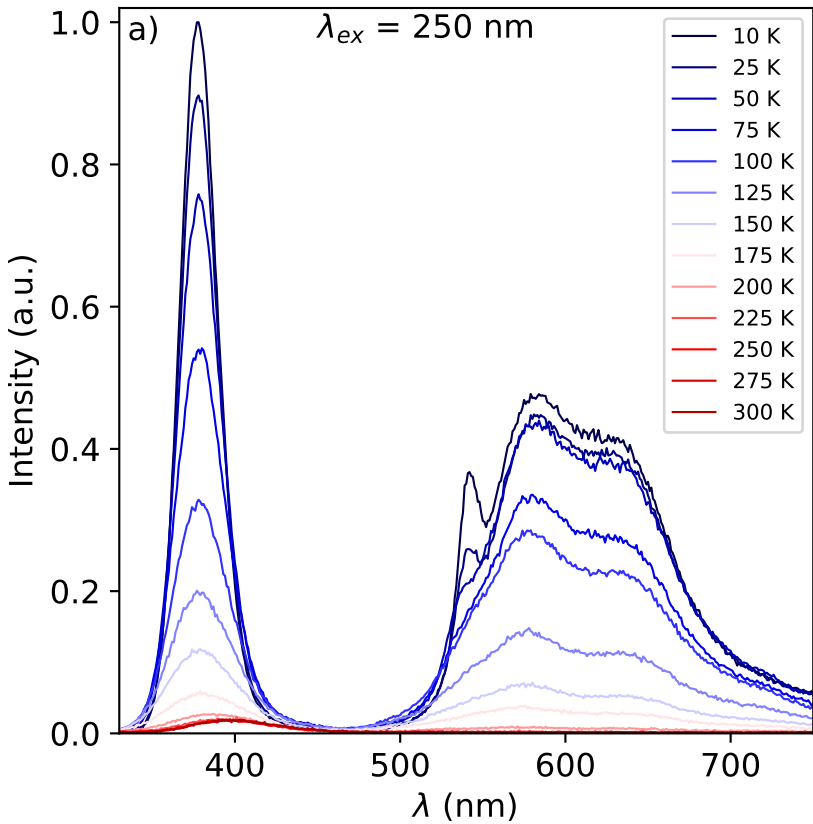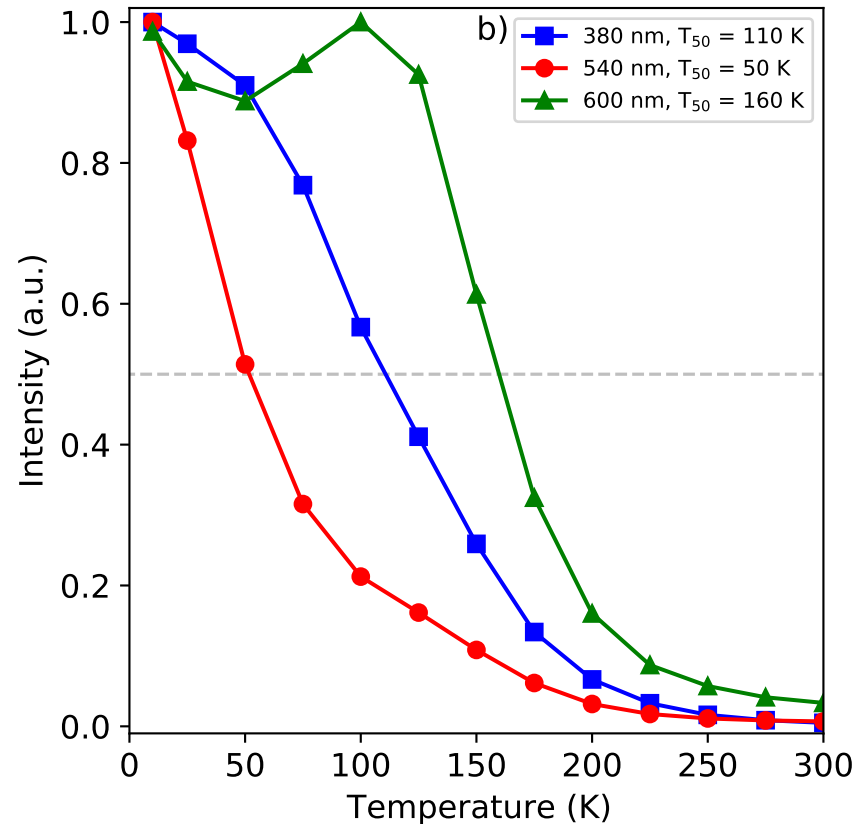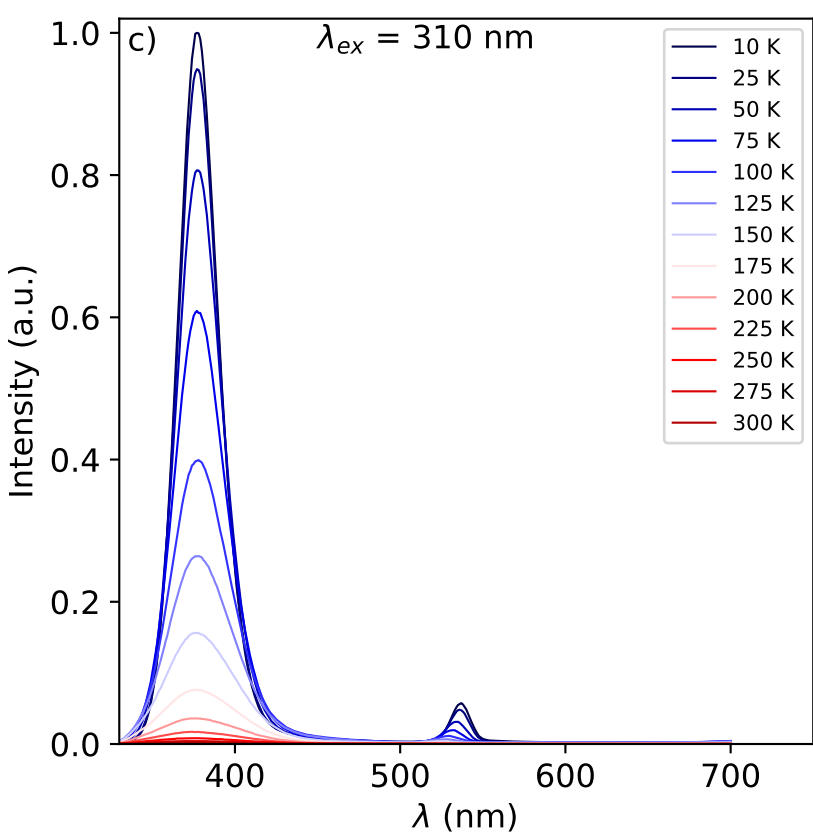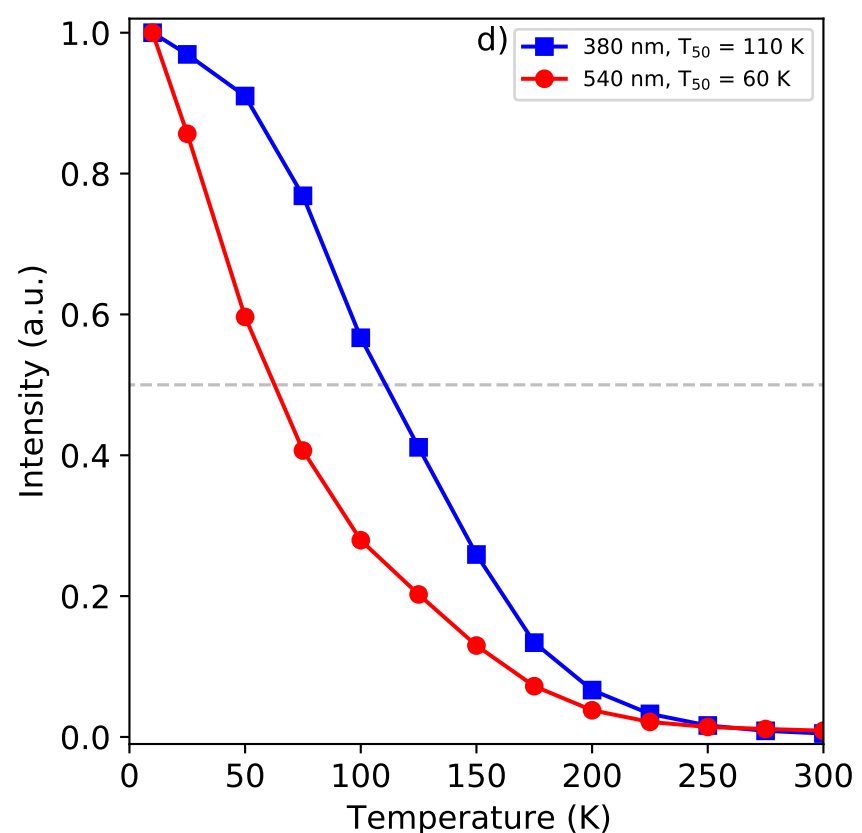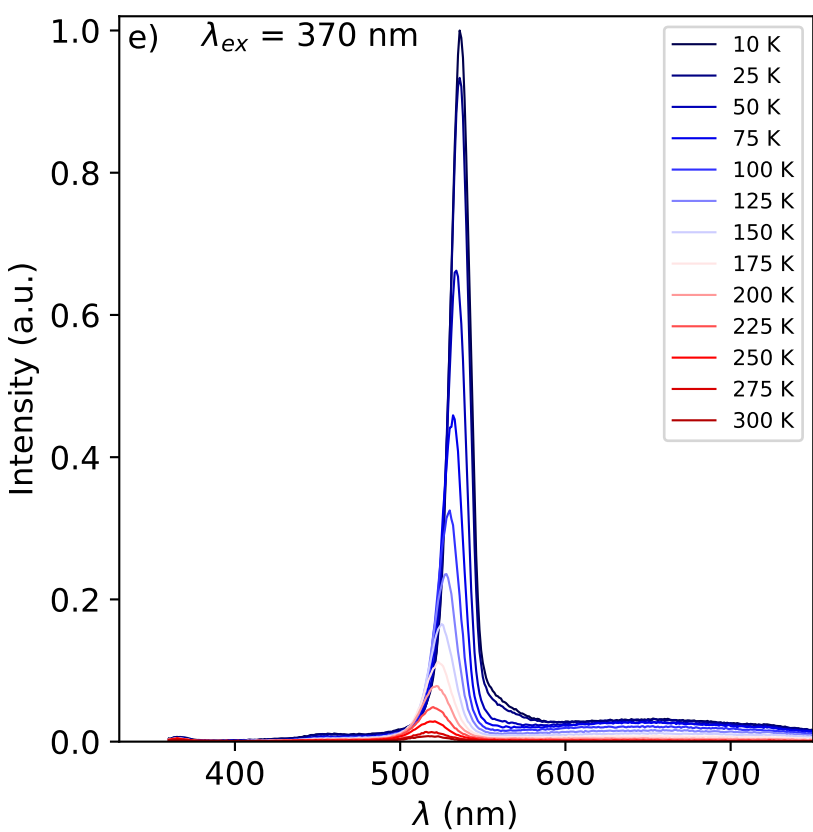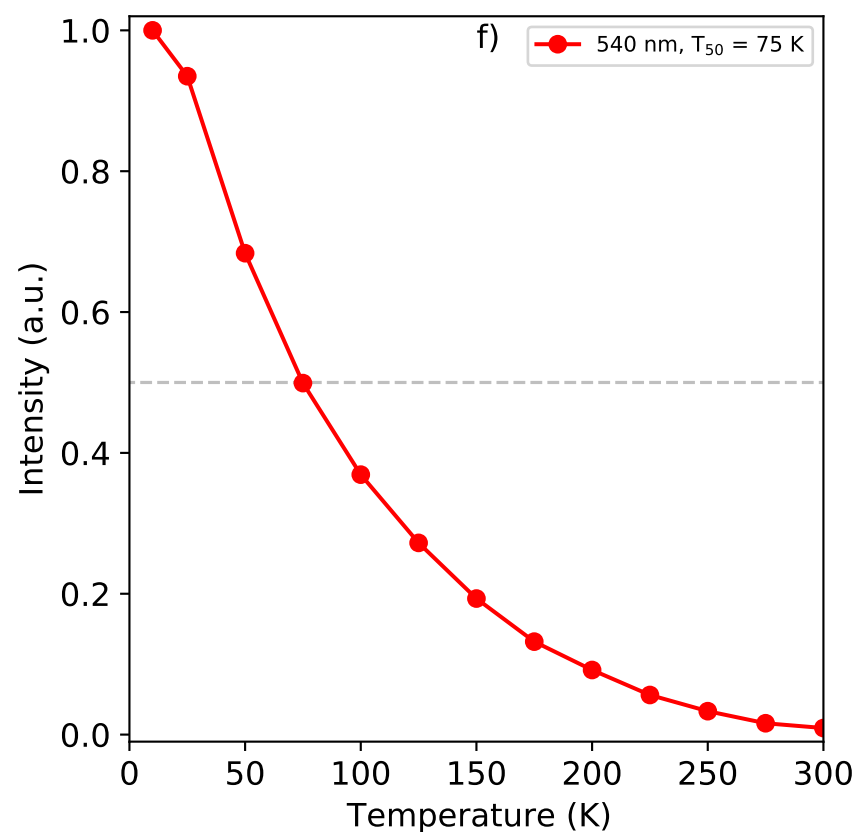

Supplement: Supplementary file 1 — jp4c06347_si_001.zip [file jp4c06347_si_001.zip › PL_TD.pdf]

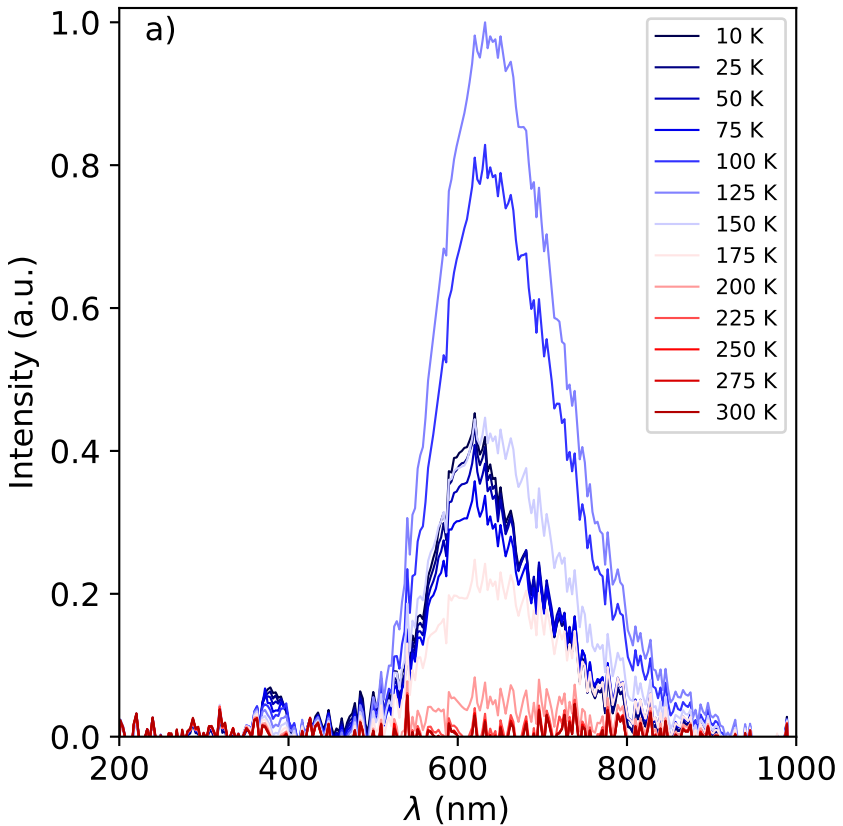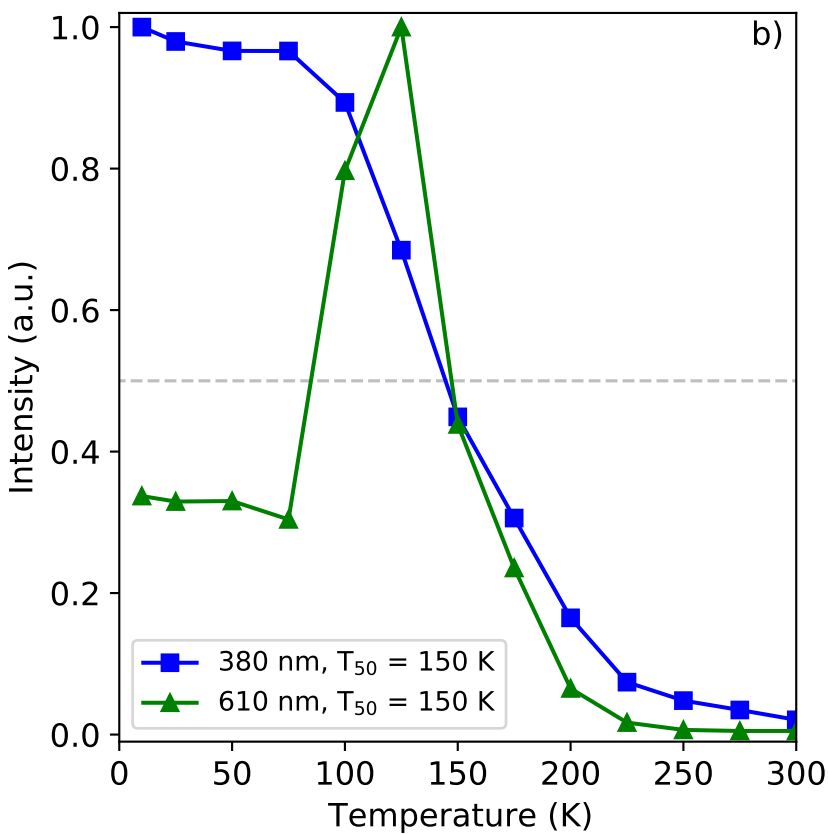

Supplement: Supplementary file 1 — jp4c06347_si_001.zip [file jp4c06347_si_001.zip › Cs4PbBr6_TDRL_p.pdf]

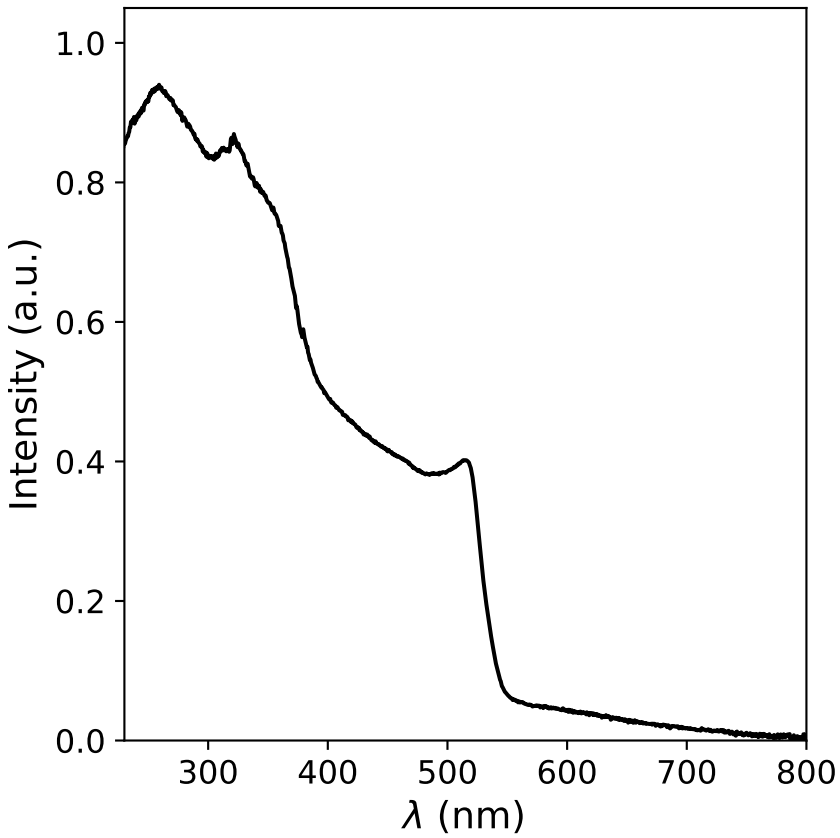

Supplement: Supplementary file 1 — jp4c06347_si_001.zip [file jp4c06347_si_001.zip › CsPbBr3_abs.pdf]

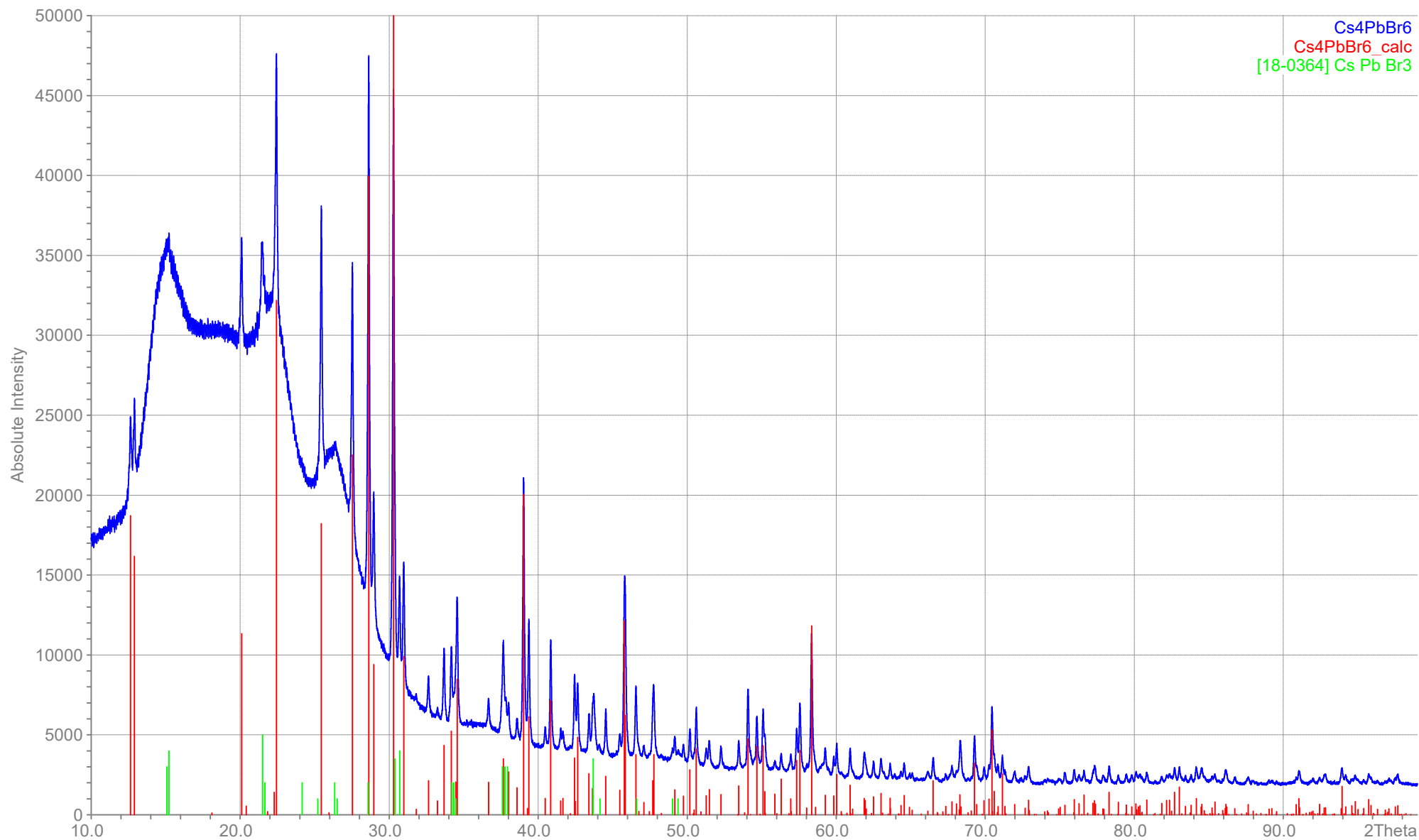

Supplement: Supplementary file 1 — jp4c06347_si_001.zip [file jp4c06347_si_001.zip › Cs4PbBr6_XRD_sc.pdf]

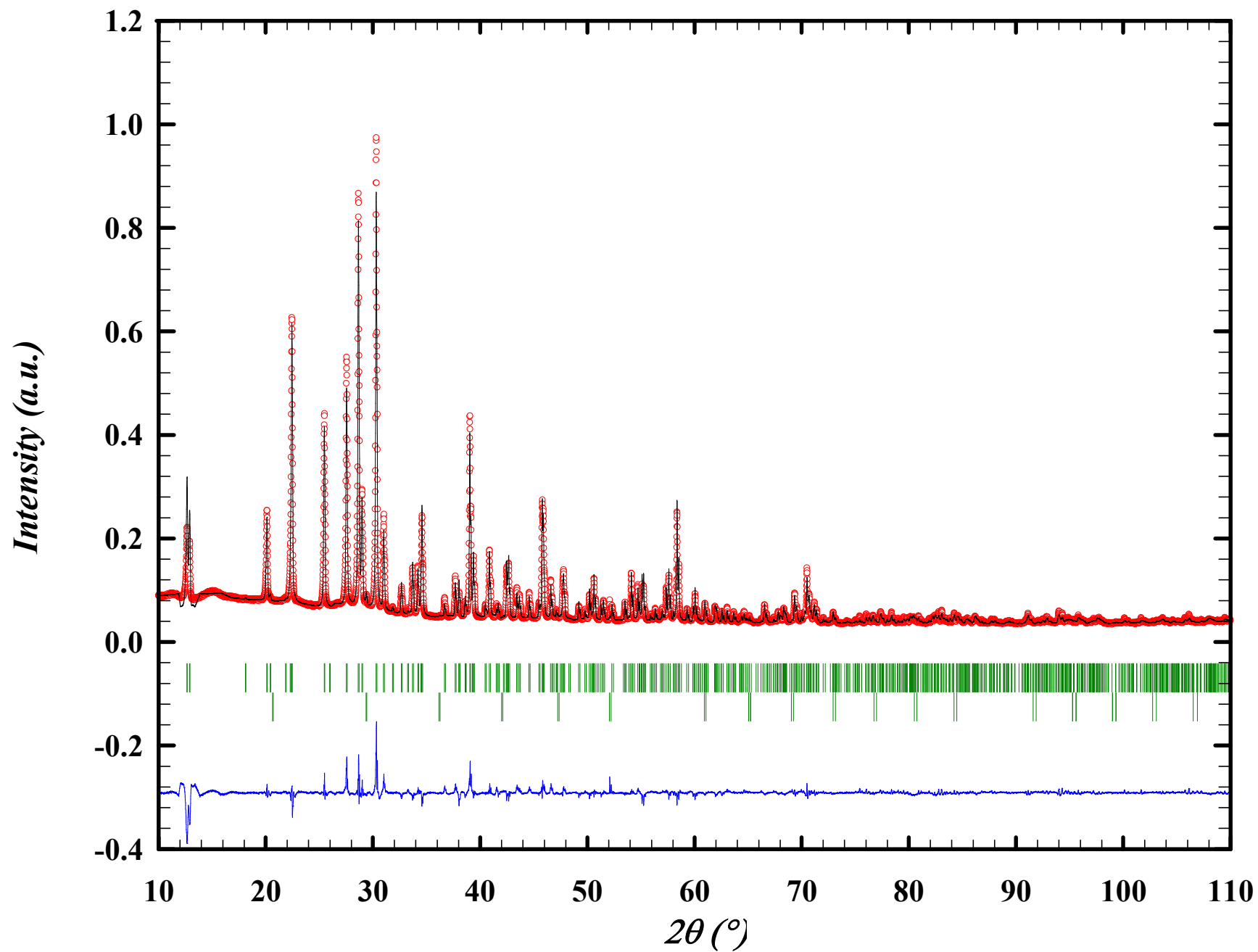

Supplement: Supplementary file 1 — jp4c06347_si_001.zip [file jp4c06347_si_001.zip › Cs4PbBr6_XRD_p.pdf]

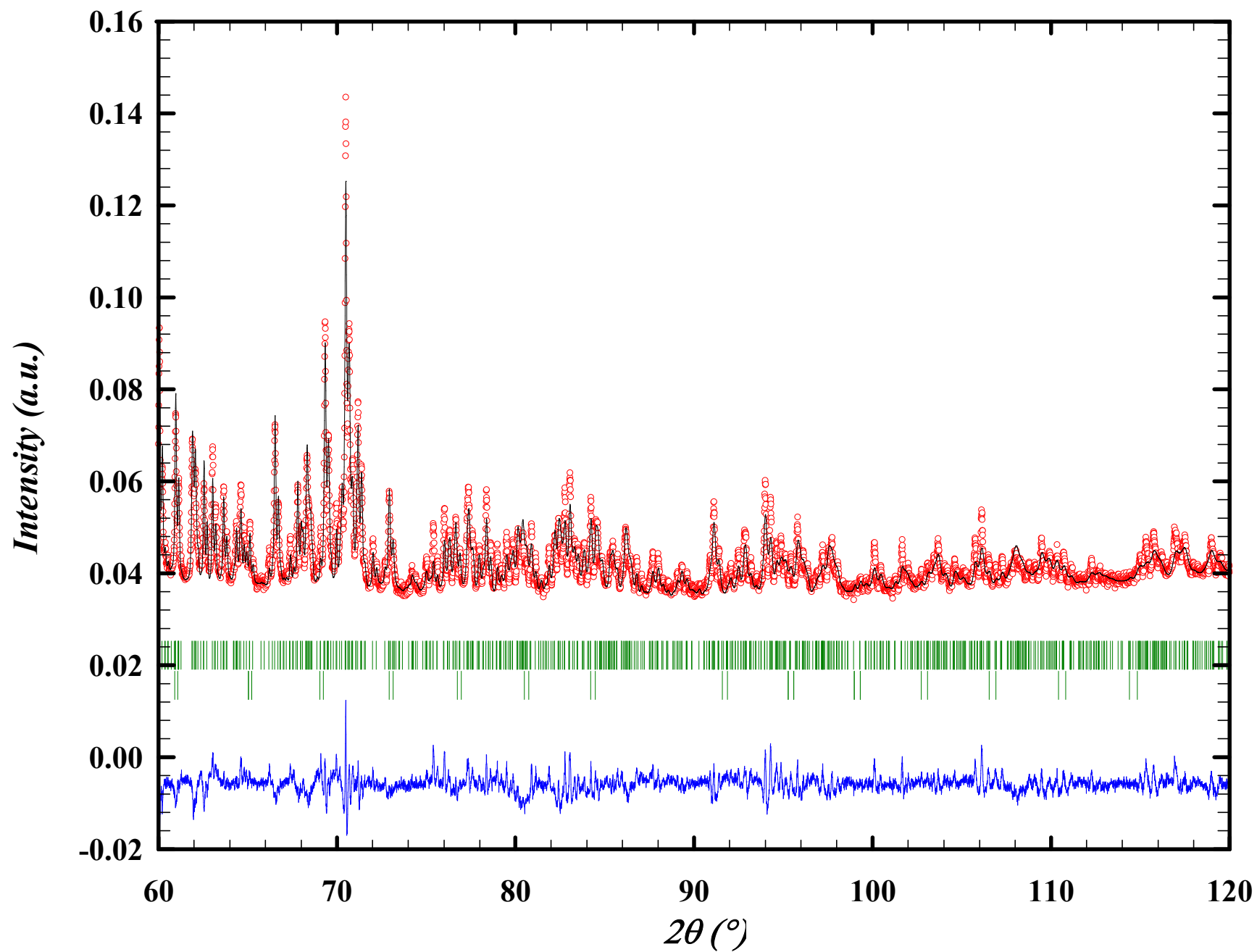

Supplement: Supplementary file 1 — jp4c06347_si_001.zip [file jp4c06347_si_001.zip › Cs4PbBr6_XRD_p_zoom.pdf]
