# Supplementary material for: Photoluminescence and Scintillation Mechanism of Cs4PbBr6
Source: J Phys Chem C Nanomater Interfaces. 2024 Nov 12;128(46):19921–32. doi: 10.1021/acs.jpcc.4c06347 (PMC11587101; doi:10.1021/acs.jpcc.4c06347)
Supplement: Supplementary file 2 — jp4c06347_si_002.pdf [file jp4c06347_si_002.pdf]

The Photoluminescence and Scintillation Mechanism of Cs_4PbBr_6 Supplementary Information

J. Jasper van Blaaderen[1], Andries van Hattem[1], Jence T. Mulder[2], Daniel Biner[3], Karl W. Krämer[3], Pieter Dorenbos[1]

1: Delft University of Technology,

Faculty of Applied Sciences,

Department of Radiation Science and Technology,

Mekelweg 15, 2629 JB Delft,

The Netherlands

2: Delft University of Technology,

Faculty of Applied Sciences,

Optoelectronic Materials Section,

Van der Maasweg 9,

2629 HZ Delft, The Netherlands

3: Univeristy of Bern,

Department of Chemistry, Biochemistry,

and Pharmaceutical Sciences,

Freiestrasse 3, Bern, Switzerland

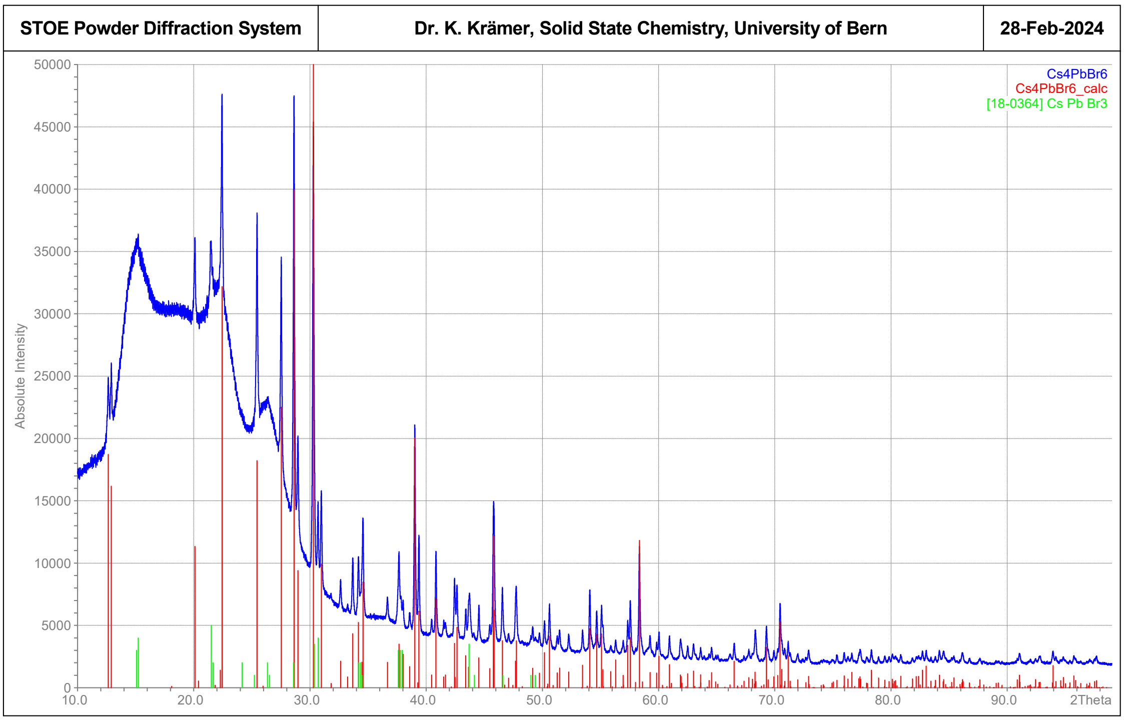

FIG. S1: Powder diffraction pattern of Cs_4PbBr_6 with about 10% $CsPbBr_3$ inclusions measured with Cu $K_{\alpha 1}$ radiation at room temperature. The three broad peaks below 30° 2-Theta originate from the Mylar window of the sample holder.

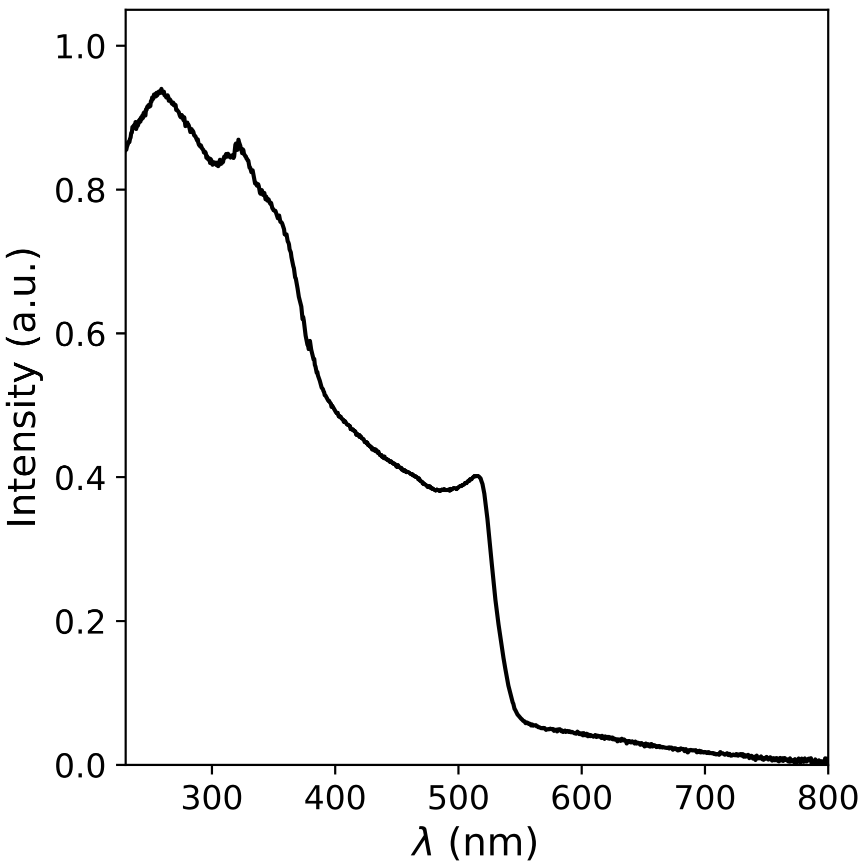

FIG. S2: Room temperature absorbance spectrum of the $CsPbBr_3$ single crystal.

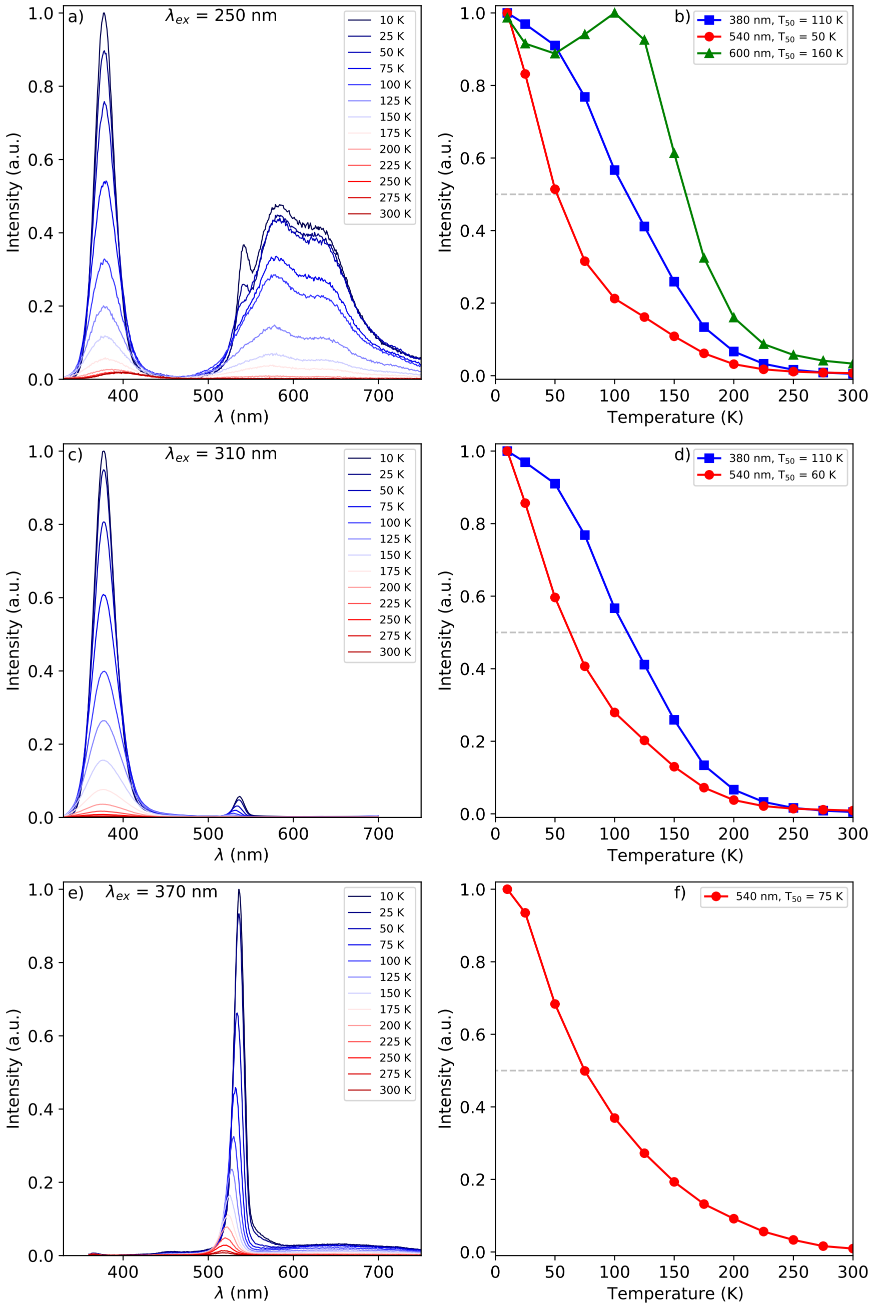

FIG. S3: Temperature dependent photoluminescence emission spectra of Cs_4PbBr_6 single Crystal with $CsPbBr_3$ inclusions from 10 to 300 K recorded by exciting at (a) 250 nm, (c) 310 nm, and (e) 350 nm.

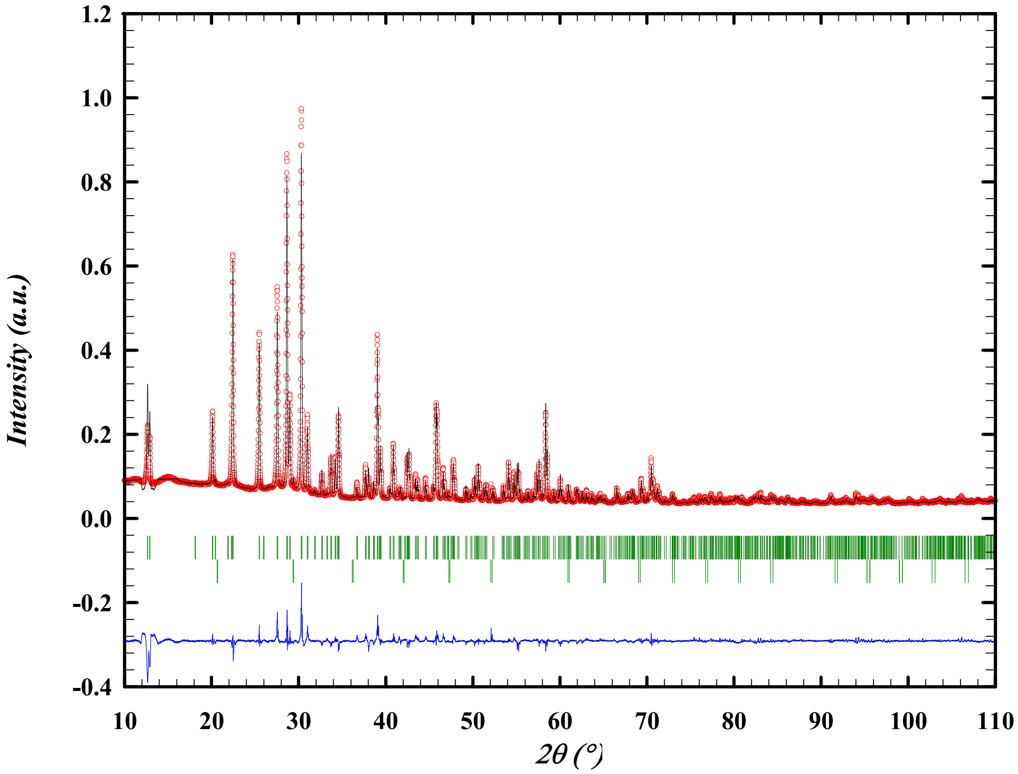

FIG. S4: Experimental ($Y_{observed}$, in red circles) and calculated ($Y_{calculated}$, in black) powder XRD patterns of synthesised Cs_4PbBr_6. The difference between calculated and experimental intensities $Y_{observed} - Y_{calculated}$ is shown in blue. The angular positions of Bragg reflections for Cs_4PbBr_6 and CsBr are shown in green. Measurement at λ = Cu K_α. Rietveld refinement of Cs_4PbBr_6 in space group $R\bar{3}c$ (167) and CsBr in $Pm\bar{3}m$ (221).

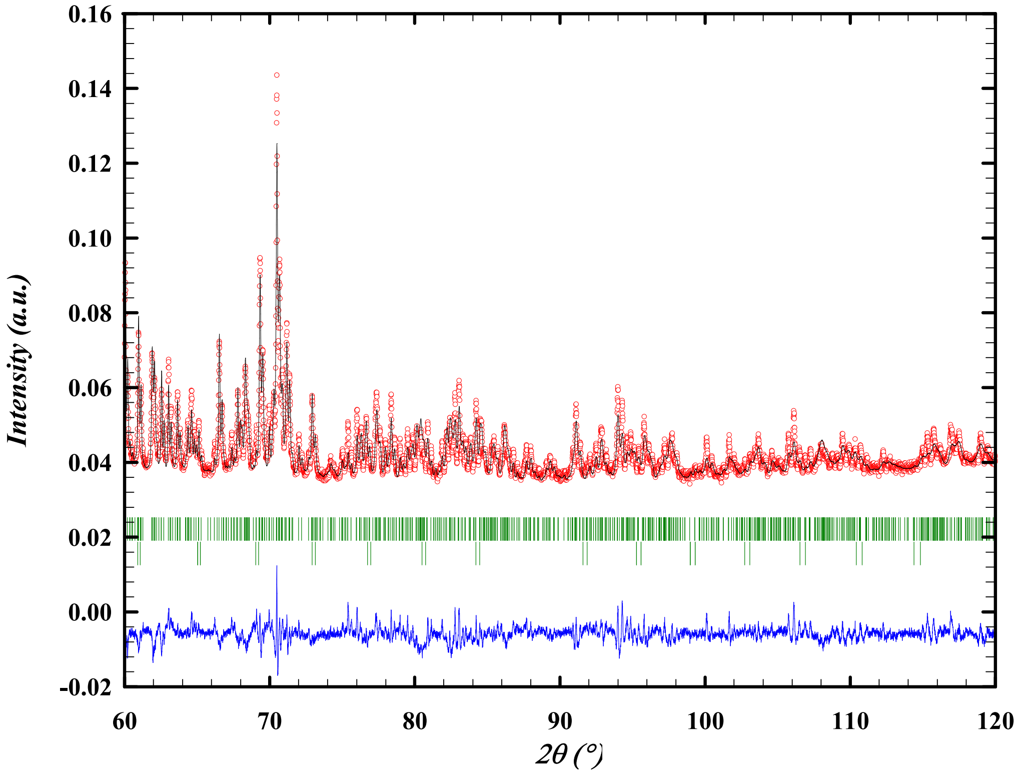

FIG. S5: Zoom in of the experimental ($Y_{observed}$, in red circles) and calculated ($Y_{calculated}$, in black) powder XRD patterns of synthesised Cs_4PbBr_6. The difference between calculated and experimental intensities $Y_{observed} - Y_{calculated}$ is shown in blue. The angular positions of Bragg reflections for Cs_4PbBr_6 and CsBr are shown in green. Measurement at λ = Cu K_α. Rietveld refinement of Cs_4PbBr_6 in space group $R\bar{3}c$ (167) and CsBr in $Pm\bar{3}m$ (221).

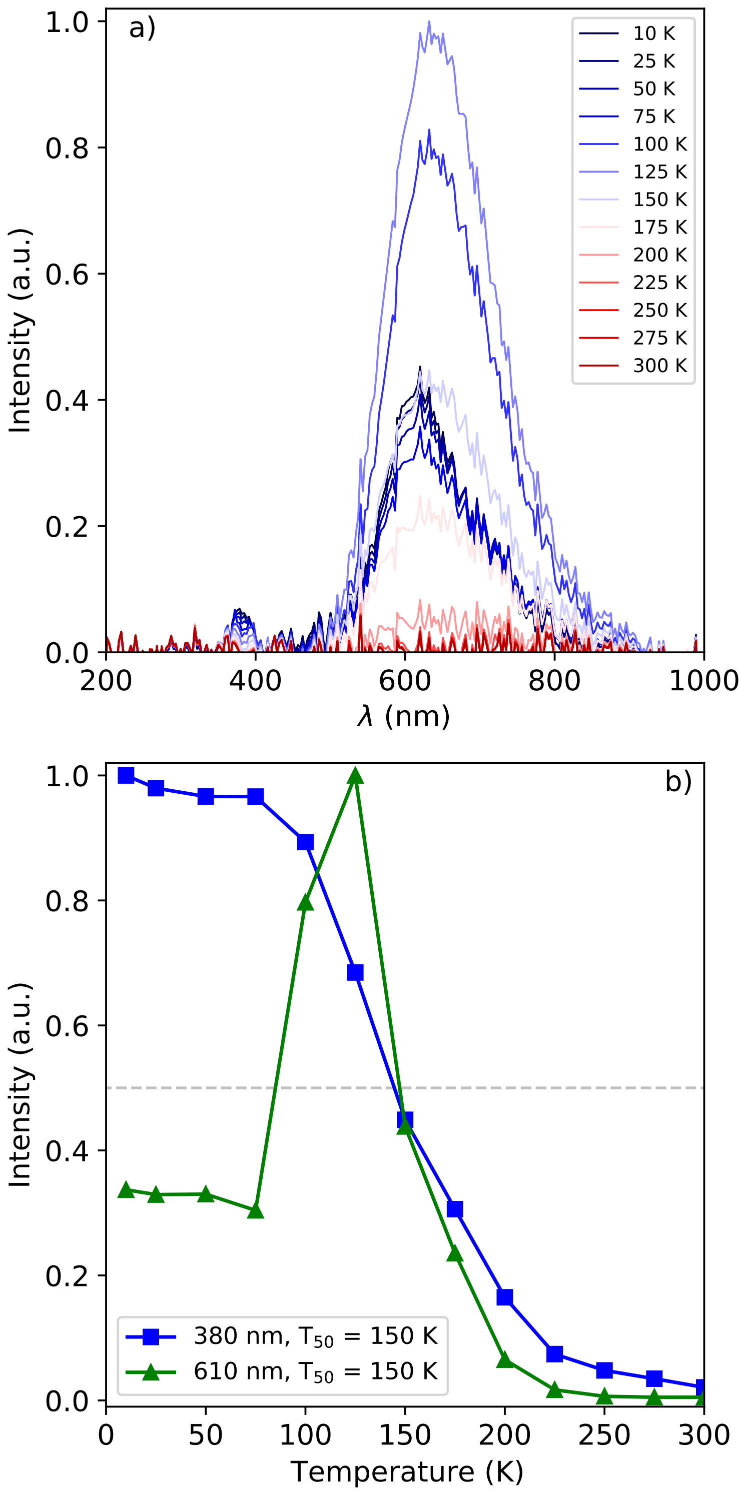

FIG. S6: (a) Temperature dependent X-ray excited emission spectra of Cs_4PbBr_6 without $CsPbBr_3$ inclusions. (b) Integrated spectral intensity.
